# Targeting a Myeloid–Regulatory B Cell Network Reverses Immune Paralysis in Periprosthetic Joint Infections

**DOI:** 10.1002/advs.76149

**Published:** 2026-06-22

**Authors:** Jintao Wu, Shutao Zhang, Yumin Lin, Juyang Jiao, Zhiwei Fu, Qimin Hong, Ziyi Zhao, Xinhua Qu, Fei Su, Bing Yue

**Affiliations:** ^1^ Department of Bone and Joint Surgery Department of Orthopedics Renji Hospital Shanghai Jiaotong University School of Medicine China; ^2^ Pediatric Orthopaedic Hospital Honghui Hospital Xi'an Jiaotong University Xi'an Shaanxi China; ^3^ Department of Endocrinology and Metabolism the First Affiliated Hospital of Nanjing Medical University Nanjing Jiangsu China; ^4^ Department of Orthopedics Jiading District Central Hospital Affiliated Shanghai University of Medicine & Health Sciences Shanghai China

**Keywords:** alendronate, immunosuppression, myeloid‐derived suppressor cells, periprosthetic joint infection, regulatory B cell

## Abstract

The localized immunosuppressive microenvironment is a primary driver of treatment failure and high recurrence in periprosthetic joint infection (PJI). Although this state of immune paralysis is critical to the persistence of infection, clinically applicable immunomodulatory strategies to reverse it remain lacking. Here, utilizing single‐cell RNA sequencing to delineate the immunosuppressive landscape of a murine PJI model, we identified a pivotal subset of polymorphonuclear myeloid‐derived suppressor cells (PMN‐MDSCs) characterized by high CXCR4 expression. Mechanistically, we reveal that these CXCR4^+^ PMN‐MDSCs interact with AHR^+^ and TIM1^+^ regulatory B cells (Bregs) to sustain a robust immunosuppressive axis. Modulation of CXCR4 signaling reduced the secretion of immunosuppressive mediators, such as ARG1, and decreased Breg abundance, effectively disrupting this suppressive network. To translate these immunological insights into clinical practice, we employed drug screening and identified alendronate as a specific inhibitor of CXCR4^+^ PMN‐MDSCs that acts directly via the signal transducer and activator of transcription 3 (STAT3). In vivo evidence from multiple infection models, combining alendronate with vancomycin significantly enhanced bacterial clearance, reversed local immune paralysis, and profoundly promoted structural bone repair. These findings uncover a druggable CXCR4^+^ PMN‐MDSC‐Breg immune tolerance network, offering a highly translational immunomodulatory paradigm for overcoming persistent skeletal infections.

## Graphical abstract

Periprosthetic joint infection establishes a sophisticated immunosuppressive network between CXCR4^+^ PMN‐MDSCs and Bregs, inducing profound CD8^+^ T cell paralysis. Alendronate effectively disrupts this CXCR4^+^ PMN‐MDSC–Breg axis by targeting STAT3, thereby restoring local immune surveillance. This therapeutic strategy facilitates bacterial clearance and promotes bone remodeling, offering a potent clinical approach to overcome chronic implant‐related infections.

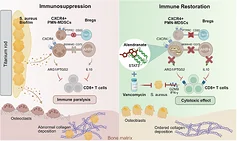

## 1 Introduction

Periprosthetic joint infection (PJI) remains a formidable complication in orthopedics, exerting a profound burden on global healthcare systems. Despite its modest incidence, the clinical impact of PJI is underscored by its staggering mortality and economic strain. Specifically, the post‐operative 5‐year mortality for knee PJI reaches 21.64% [1], while early‐onset infection within the first year elevates the decadal death risk by over five times [2]. Traditional therapeutic paradigms, including optimized antibiotic regimens and refined surgical debridement, often fall short of preventing chronic persistence or late‐stage recurrence. Such failures are frequently driven by the emergence of multidrug‐resistant pathogens like methicillin‐resistant staphylococcus aureus (MRSA), alongside sophisticated bacterial survival tactics such as biofilm architecture and intracellular sequestration. Beyond these bacterial factors, the pathogen‐induced immunosuppressive microenvironment has emerged as a critical determinant of infection outcomes. Current clinical consensus on sepsis already defines these immunosuppressive states through the functional paralysis of innate and adaptive immunity, offering a framework for diagnostic monitoring [3]. Moreover, the dynamic complexity and inherent challenges of these immune exhaustion networks throughout different infection phases have been extensively characterized [4]. Nevertheless, while immunomodulatory intervention represents a transformative antimicrobial frontier, its clinical translation in PJI is currently hindered by an incomplete mapping of the local immunosuppressive architecture.

Myeloid‐derived suppressor cells (MDSCs) are recognized as cornerstone regulators of immune evasion in malignancy. Growing evidence reveals that they orchestrate tumor progression through diverse modalities, such as paralyzing antitumor T cell surveillance, stimulating neovascularization, driving epithelial–mesenchymal transition, and priming metastatic niches, which collectively linked to unfavorable clinical prognoses across various cancers [5, 6, 7]. In contrast, the involvement of MDSCs in infectious pathologies remains an underdeveloped field of study. Hypoxia has emerged as a definitive trigger that compromises MDSC‐mediated anti‐infective immunity. Specifically, the master regulator HIF1α modulates glycolytic metabolism and the production of reactive oxygen species (ROS) within MDSCs, thereby facilitating biofilm development and elevating host vulnerability to sepsis [8, 9]. Furthermore, paralleling their role in oncology, MDSCs in infectious environments can liberate arginase‐1 (ARG1), which subsequently impairs T‐cell effector functions [10]. The immunosuppressive role of MDSCs in infectious contexts warrants significant attention and cannot be overlooked. MDSCs are divided into two main subgroups: monocytic MDSCs (M‐MDSCs) and polymorphonuclear MDSCs (PMN‐MDSCs), which differ in phenotype, inhibitory mechanisms, and therapeutic efficacy [11, 12]. PMN‐MDSCs exhibit a trend of massive expansion in both tumors and infectious diseases, resulting in a greater overall immunosuppressive burden compared to M‐MDSCs [13, 14]. PMN‐MDSCs inhibit immune‐active cells like T cells, while promoting the proliferation and migration of cells that compromise immune defense, such as regulatory T cells (Tregs) and tumor cells, ultimately accelerating disease progression and worsening prognosis [15, 16]. Therefore, deepening comprehension of the pathways through which PMN‐MDSCs exert immunosuppression and identifying suitable therapeutic targets is of great significance for improving disease outcomes and preventing relapse.

The biological revolution driven by omics technologies has redefined the landscape of clinical research, steering it toward the frontier of precision medicine. This paradigm shift emphasizes that dissecting disease‐specific cellular subpopulations is fundamental for refined molecular stratification and the identification of high‐fidelity therapeutic targets. Recently, distinct PMN‐MDSC clusters characterized by elevated expression of CD84, CD14, CD52, or PTGER2 have been documented within various oncological environments [17, 18, 19]. However, detailed descriptions and analyses of PMN‐MDSC subtypes in infectious diseases, particularly in the context of PJI, remain scarce. Moreover, current research on PMN‐MDSCs in infectious diseases predominantly extrapolates findings from tumor studies, such as their role in inhibiting T cell function. This approach overlooks the intricate regulatory network of immune suppression. Regulatory B cells (Bregs), characterized by the secretion of interleukin‐10 (IL‐10) and other inhibitory cytokines, have been reported to participate in the development of infectious diseases. In tuberculosis, for example, Bregs induce T cell apoptosis and tolerance through the secretion of Fas ligand [20]. Furthermore, Bregs would suppress anti‐infective immunity by secreting relevant factors in infections caused by Escherichia coli, Helicobacter pylori, and Chlamydia trachomatis [21, 22, 23]. Whether a functional synergy exists between PMN‐MDSCs and Bregs—two pivotal immunosuppressive populations—within the infectious milieu, and how their potential interplay structures the inhibitory landscape of PJI, constitutes a critical knowledge gap that warrants urgent exploration.

In silico drug discovery has substantially enhanced the efficiency of drug discovery. Nevertheless, when targeting specific cell subtypes, drug screening strategies based solely on their highly expressed genes frequently fail to achieve precise targeting. Although numerous algorithms have been developed for screening drugs in single‐cell subpopulations, the majority of them predominantly offer guidance for tumor therapy [24]. In this context, the Scissor algorithm offers a distinct advantage by integrating drug sensitivity profiles from bulk transcriptomics with single‐cell datasets, thereby pinpointing highly responsive cellular subpopulations [25]. This approach undoubtedly significantly improves the applicability and precision of drug screening. Alendronate, a second‐generation bisphosphonate, is clinically utilized to treat osteoporosis due to its capacity to inhibit osteoclast activity and reduce bone resorption [26]. However, recent studies have revealed that bisphosphonates, including alendronate, exhibit therapeutic potential against infectious conditions. The use of alendronate or zoledronate alone can directly suppress the proliferation of Cryptococcus and various fungi. When combined with fluconazole, these agents can markedly reduce drug dosage and effectively inhibit biofilm formation [27, 28]. Recently, bisphosphonates, particularly alendronate, have been employed to construct targeted delivery systems aimed at alleviating bone infections, indicating the potential of alendronate in treating infectious bone diseases [29, 30]. Furthermore, alendronate has demonstrated the capacity to amplify the effectiveness of antimicrobial agents in treating osteomyelitis, though the specific mechanism remains unclear [31]. Currently, it remains uncertain whether alendronate is effective for PJI, and our understanding of its synergistic anti‐infective mechanism is still very limited.

Driven by the aforementioned clinical hurdles, we constructed a Staphylococcus aureus (S. aureus)‐induced PJI model and conducted high‐resolution single‐cell RNA sequencing (scRNA‐seq). Through comprehensive immune landscape analysis and identification of MDSC characteristics, we discovered a PMN‐MDSC subtype with high expression levels of CXCR4, which is prevalent in bacterial infections and is associated with a poor prognosis. Furthermore, CXCR4^+^ PMN‐MDSCs can modulate Bregs, and together they form an immunosuppressive network in PJI. Inhibition of CXCR4 effectively reduces CXCR4^+^ PMN‐MDSCs and Bregs, thereby alleviating PJI. Alendronate has been identified as an effective targeted drug for CXCR4^+^ PMN‐MDSCs, and its combination with vancomycin has demonstrated favorable therapeutic effects in both skin infections and PJI by effectively mitigating the immunosuppressive microenvironment. In summary, this study identified a CXCR4^+^ PMN‐MDSC subtype associated with bacterial infection and screened for effective targeted therapeutic drugs, offering novel insights for the diagnosis and treatment of PJI.

## 2 Result

### 2.1 PMN‐MDSCs Play a Key Role in Immunosuppression Throughout the Different Stages of PJI

**FIGURE 1:**
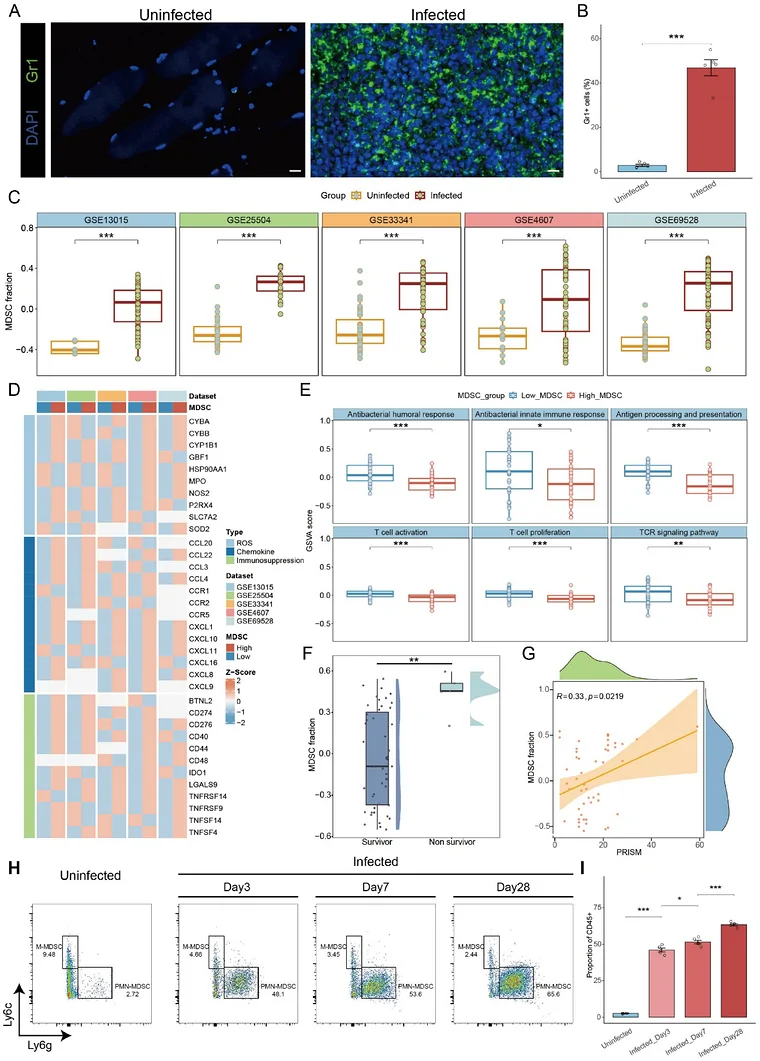
PMN‐MDSCs Play a Key Role in Immunosuppression throughout the Different Stages of PJI. (A, B) Representative immunofluorescence images and statistical analysis of Gr1^+^ cells in knee joint soft tissue of mice in the indicated groups (n = 5 per group, scale bar: 10 µm). (C) The MDSC fraction between the uninfected and infected groups in different datasets. (D) A heatmap of z‐score expression for ROS, chemokine, and immunosuppressive genes in high‐ and low‐MDSC groups across datasets. (E) GSVA score of anti‐bacterial‐related pathway between the uninfected and infected groups in GSE13015. (F) The MDSC fraction between the survivor and non‐survivor groups in GSE4607. (G) Correlation analysis between the MDSC fraction and PRISM score in GSE4607. (H, I) FC analysis was conducted on PMN‐MDSC and M‐MDSC in the knee joint soft tissue of PJI mice on days 3, 7, and 28.; statistical analysis regarding PMN‐MDSC proportion (n = 5 per group). (Bar plot displays the means ±SD; Box plot displays the mean value; *p < 0.05, ***p < 0.001.).

S. aureus stands out as the most prevalent pathogen in PJI. Immunofluorescence analysis revealed a significant accumulation of MDSCs in the infected regions of S. aureus PJI (Figure 1A,B). To investigate the relationship between bacterial infection and MDSC, an MDSC signature was scored in different bulk RNA datasets. The infected group sample presented a significantly higher amount of MDSCs (Figure 1C). Based on the median value of the MDSC fraction, the samples were categorized into high and low MDSC groups. It was observed that genes associated with inflammation and immune suppression, such as NOS2, CCL4, and CD274, exhibited higher expression levels in the high MDSC group (Figure 1D). MDSCs appear to interfere with anti‐infective immunity, as evidenced by the significantly reduced activity of antimicrobial infection pathways in the high MDSC group (Figure 1E; Figure S1A–D). These pathways include antibacterial immunity, antigen presentation, and T cell function, among others. This finding aligns with previous literature indicating that MDSCs inhibit T cell function. Importantly, MDSC abundance is correlated with the prognosis of bacterial infections. Patients who survived and those with lower PRISM risk scores exhibited a reduced abundance of MDSCs, suggesting a potential association between MDSCs and the progression of bacterial infections (Figure 1F,G).

PMN‐MDSCs and M‐MDSCs are known to be two distinct subtypes of MDSCs. To determine which subtype plays a more pivotal role in PJI, we established PJI models at three different time points: day 3, day 7, and day 28, corresponding to the acute, subacute, and chronic phases of PJI infection, respectively. FC results showed that the proportion of PMN‐MDSCs in PJI mice was notably higher compared to the control group. As the infection progressed, the proportion of PMN‐MDSCs progressively increased, accounting for 65.6% of CD45^+^ cells by day 28 post‐infection. Conversely, the corresponding proportion of M‐MDSCs gradually declined (Figure 1H,I; Figure S1E). By separately evaluating the fractions of PMN‐MDSCs and M‐MDSCs in the bacterial infection dataset GSE4607, which includes survival information, we discovered that only PMN‐MDSCs were significantly associated with the prognosis of bacterial infection (Figure S1F,G). Collectively, these findings suggest that PMN‐MDSCs may play a more pivotal role in the onset and progression of PJI.

### 2.2 PMN‐MDSCs in PJI Exhibit Characteristic High Expression of CXCR4

**FIGURE 2:**
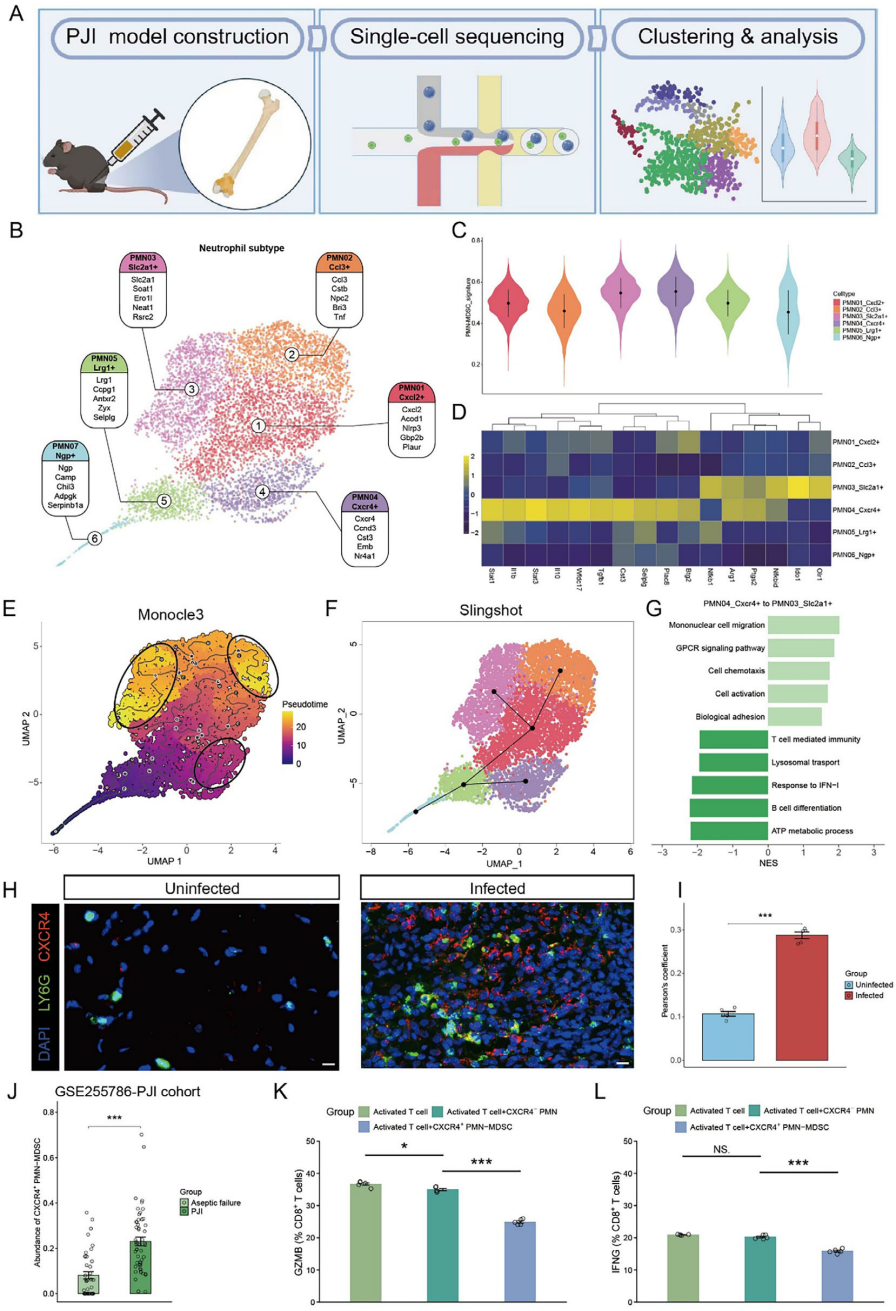
PMN‐MDSCs in PJI Exhibit Characteristic High Expression of CXCR4. (A) Flow chart of PJI model construction and scRNA‐seq (Created in https://BioRender.com). (B) UMAP plot and marker gene dot plot for PMN subtypes. (C) Violin plot for the PMN‐MDSC signature in PMN subtypes. (D) A heatmap displaying the z‐score expression levels of classical PMN‐MDSC genes across different PMN subtypes. (E,F) Monocle3 and slingshot analyses present the differentiation trajectory among PMN subtypes. (G) GOBP analysis comparing PMN04_CXCR4^+^ and PMN03_SLC2A1^+^. (H,I) Representative immunofluorescence images and statistical analysis of CXCR4^+^ PMN‐MDSCs in the knee joint soft tissue of mice in the indicated groups. (n = 5 per group, Scale bar: 10 µm.) (J) Estimation of CXCR4^+^ PMN‐MDSC abundance differences in the human PJI cohort GSE255786 using deconvolution algorithms (AF = 40, PJI = 53). (K,L) Statistical analysis of GZMB and IFNG secretion ratio in activated T cells co cultured with CXCR4^−^ PMN or CXCR4^+^ PMN‐MDSC (n = 5 per group). (Box plot displays the mean value; ns = not significant, * *p* < 0.05, ***p < 0.001.).

Due to the notably higher proportion of PMN‐MDSCs observed in day 28 of infection, we established a 28‐day PJI mouse model. Subsequently, soft tissues surrounding the knee joint were collected and scRNA sequencing was conducted to elucidate the distinctive characteristics of PMN‐MDSCs in PJI (Figure 2A). Prior to this, we commenced by delineating the immune landscape of PJI. After rigorous data quality control, all cells were systematically annotated and categorized into 11 distinct cell types, including neutrophils (S100a8, S100a9, Retnlg), B cells (Cd79a, Cd79b, Ms4a1), T cells (Cd3e, Cd3g, Trbc2), macrophages (Lyz2, Apoe, Cd68), fibroblasts (Dcn, Col1a1, Col1a2), natural killer (NK) cells (Nkg7, Ncr1, Gzmb), plasma cells (Jchain, Mzb1, Igkc), endothelial cells (Pecam1, Cdh5, Cd36), classical dendritic cell (cDCs) (Fscn1, H2‐Aa, H2‐Eb1), smooth muscle cells (SMCs) (Acta2, Rgs5, Tagln), and mast cells (Cma1, Cpa3, Kit) (Figure S2A,B). In terms of overall cellular composition, the proportion of neutrophils in PJI‐infected mice exhibited a rapid increase, whereas the proportions of T cells and fibroblasts declined notably (Figure S2C). These patterns imply the potential presence of an overactivated inflammatory response, coupled with diminished anti‐infective immunity and impaired tissue repair mechanisms during PJI infection.

To further identify PMN‐MDSCs, we extracted neutrophils for secondary dimensionality reduction clustering. With a resolution setting of 0.8, a total of six distinct PMN subtypes were identified, each exhibiting high expression levels of Cxcl2, Ccl3, Slc2a1, Cxcr4, Lrg1, and Ngp, respectively (Figure 2B, Figure S2D). The stacked bar chart results indicated that PMN01_Cxcl2^+^, PMN02_Ccl3^+^, PMN03_Slc2a1^+^, and PMN04_Cxcr4^+^ displayed varying degrees of increase in the infected group (Figure S2E). Three independent PMN‐MDSC gene sets were employed to identify PMN‐MDSCs in PJI. The results demonstrated that both PMN03_Slc2a1^+^ and PMN04_Cxcr4^+^ exhibited significantly higher scores compared to other subgroups (Figure 2C; Figure S2F,G). These two cell populations jointly overexpressed certain PMN‐MDSC marker genes, such as Arg1 and Ptgs2, albeit with differing expression patterns. PMN03_Slc2a1^+^ specifically displayed heightened expression of Ido1, and was associated with the NFKB pathway. In contrast, PMN04_Cxcr4^+^ expressed higher levels of Il1b and was linked to the STAT transcription factor family. These distinct expression patterns underscored the heterogeneity of PMN‐MDSCs in PJI (Figure 2D).

Monocle3 and Slingshot analyses have elucidated the differentiation trajectory of PMN subtypes. Given the expression of multiple neutrophil precursor cell markers (Ngp, Camp, Chil3) in the PMN07_Ngp^+^ subtype, it was positioned at the origin of the differentiation trajectory, serving as a basis for inferring the overall differentiation outcomes. PMN02_Ccl3^+^, PMN03_Slc2a1^+^, and PMN04_Cxcr4^+^ were observed to represent three distinct endpoints of differentiation (Figure 2E,F). Notably, PMN04_Cxcr4^+^ was found to be at an earlier stage of differentiation compared to PMN03_Slc2a1^+^. To assess which PMN‐MDSC subtype is more prone to suppress antibacterial immunity, we performed differential expression and enrichment analyses between PMN03_Slc2a1^+^ and PMN04_Cxcr4^+^. The results indicated that PMN04_Cxcr4^+^ exhibited more pronounced adaptive immune suppression than PMN03_Slc2a1^+^, affecting both T cells and B cells (Figure 2G). Concurrently, PMN04_Cxcr4^+^ downregulated multiple antibacterial and immune processes, such as the response to IFN I. The immunofluorescence results indicate that there are indeed a large number of CXCR4^+^ PMN‐MDSCs are present in the PJI infected area (Figure 2H,I). Concurrently, we evaluated the abundance of CXCR4^+^ PMN‐MDSCs using the human PJI cohort GSE255786. Consistent with our findings in mouse soft tissues, the PJI group exhibited a significantly higher proportion of CXCR4^+^ PMN‐MDSCs compared to the AF group (Figure 2J). Besides, scoring results for various neutrophil functions also revealed that PMN04_Cxcr4^+^ displayed high levels of inflammatory response, chemotaxis, and glycolysis, yet exhibited weak phagocytic capacity (Figure S2H).

To functionally validate whether the CXCR4^+^ subpopulation in PJI acts as bona fide PMN‐MDSCs, we sorted CXCR4^+^ PMN‐MDSCs and other PMN (CXCR4^−^ PMN), and performed a co‐culture assay with activated T cells. While T cell suppression is a hallmark of PMN‐MDSCs, only the CXCR4^+^ subset exhibited a robust inhibitory effect, significantly reducing GZMB and IFNG secretion (Figure 2K,L, Figure S3A). Similarly, the CXCR4^+^ subpopulation exhibited a more pronounced inhibitory effect on T cell proliferation compared to other PMN subsets (Figure S3B). Consequently, we deduce that PMN04_Cxcr4^+^ may be the key immunosuppressive cell contributing to PJI infection.

### 2.3 CXCR4^+^ PMN‐MDSCs are Widely Detected During Bacterial Infections and Correlated With Prognosis

Based on the aforementioned findings, we successfully identified CXCR4^+^ PMN‐MDSCs in PJI mice. However, it remained uncertain whether this specific cell subtype was exclusive to PJI or prevalent across various bacterial infections. To resolve this question, we obtained scRNA sequencing data from patients with sepsis, as well as from mouse models infected with E. coli and P. aeruginosa. The results revealed the presence of PMN subtypes with elevated CXCR4 expression in different infections (such as cluster 3 in sepsis, cluster 1 in E. coli infection, and cluster 3 in P. aeruginosa infection) (Figure S4A–C). Notably, this CXCR4^+^ cell population also exhibited heightened expression of PMN‐MDSC marker genes, including IL10, PTGS2, CST3, and CXCR2 (Figure S4D–F).

To further substantiate the existence of CXCR4^+^ PMN‐MDSCs, we employed flow cytometry to sort Gr1^+^ cells and conducted scRNA sequencing. The PMN‐MDSCs were annotated and subjected to secondary dimensionality reduction clustering, yielding 10 distinct PMN subtypes (Figure S4G). As anticipated, we identified PMN subtypes with high expression levels of Cxcr4 and Slc2a1 (PMN04 and PMN02), which also displayed the highest PMN‐MDSC scores (Figure S4H–J). Consequently, we have once again verified the presence of CXCR4^+^ PMN‐MDSCs.

Furthermore, we integrated five bulk datasets encompassing bacterial species information and eliminated batch effects to expand the sample size (Figure S5A,B). To our delight, we discovered that 11 out of 12 bacteria‐induced infections resulted in a significantly higher proportion of CXCR4^+^ PMN‐MDSCs than the control group (Figure S5C). Within the merged dataset, it also became evident that a high abundance of CXCR4^+^ PMN‐MDSCs was linked to an unfavorable prognosis in bacterial infections (Figure S5D,E). Our analysis of sepsis data revealed that CXCR4^+^ PMN‐MDSCs were significantly enriched in patients who had succumbed to the illness across all datasets, and exhibited a marked positive correlation with SOFA scores (Figure S5F,G). Overall, our analysis unequivocally demonstrates that CXCR4^+^ PMN‐MDSCs are widely distributed in bacterial infections and is intricately associated with survival prognosis.

### 2.4 CXCR4^+^ PMN‐MDSCs Interact With Breg Cells to Form an Immunosuppressive Network in PJI

PMN‐MDSCs are immunosuppressive cells that exhibit critical functions in bacterial infections. Within the immune microenvironment, intercellular communication serves as a crucial mechanism for cellular function. Understanding how CXCR4^+^ PMN‐MDSCs regulate and collaborate with other cells to exacerbate PJI infection represents a critical issue. Cell communication analysis conducted using the Cellchat package revealed that, although the number of communications differs between infected and uninfected cells, the communication intensity for each cell type in the infected group is significantly stronger than that in the control group in terms of both incoming and outgoing signals (Figure S6A,B). For CXCR4^+^ PMN‐MDSC, both input and output signal strengths were elevated in the infected group (Figure S6C). Among the interacting cell types, fibroblasts exhibited the strongest input signal toward CXCR4^+^ PMN‐MDSCs, while CXCR4^+^ PMN‐MDSCs demonstrated the strongest output signal toward B cells (Figure S6D).

According to prior literature, CXCL12 serves as a classic ligand for CXCR4, capable of accelerating tumor proliferation, migration, and metastasis in neoplastic contexts. Notably, we observed high specific expression of CXCL12 in fibroblasts (Figure S6E). Furthermore, the Cxcl12‐Cxcr4 LRs, which acts upon CXCR4^+^ PMN‐MDSCs during infection, received nearly the highest score in our analysis (Figure S6F). Considering that the Cxcl12‐Cxcr4 axis represents a vital pathway through which fibroblasts regulate CXCR4^+^ PMN‐MDSCs.

**FIGURE 3:**
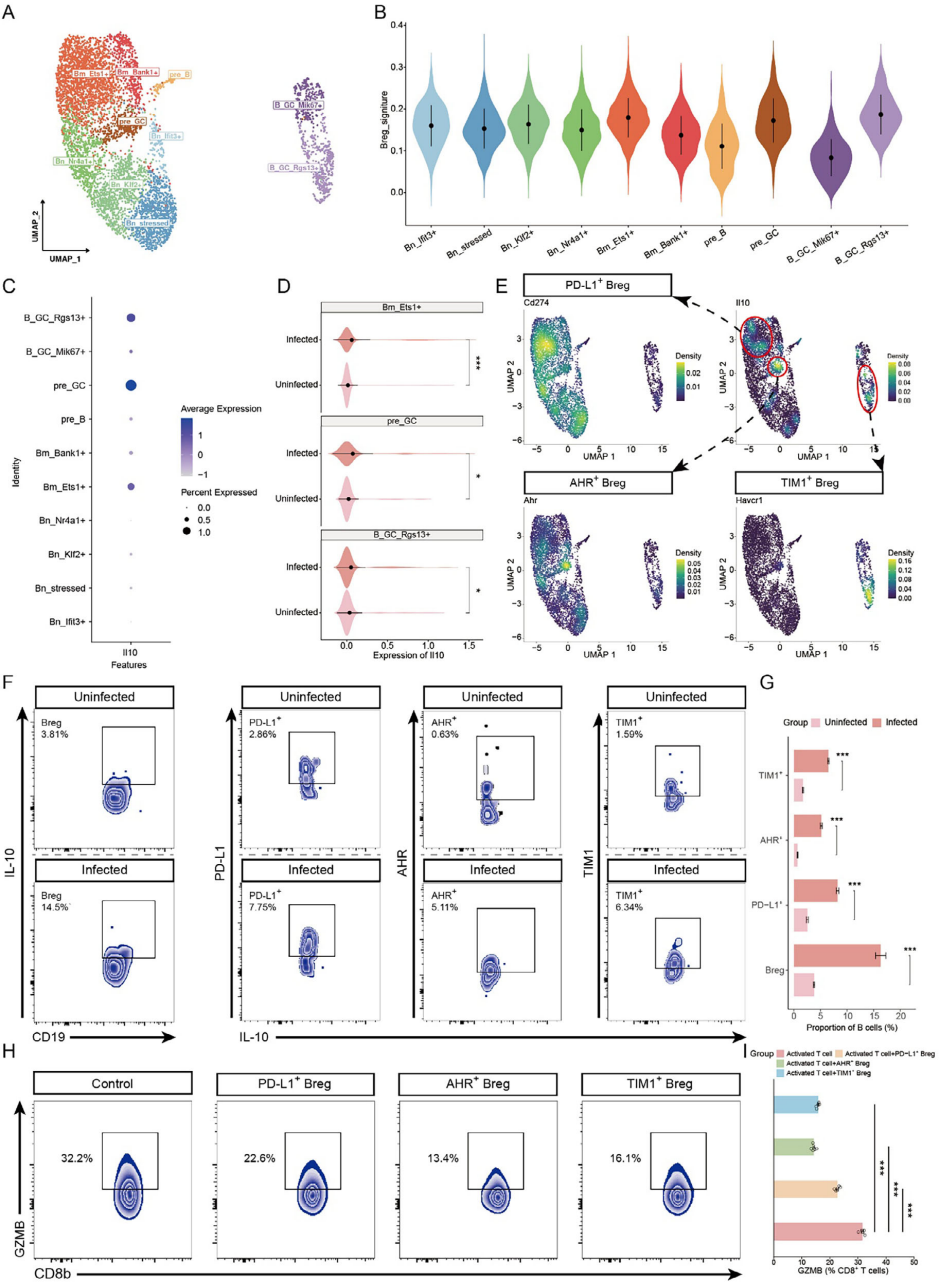
Identification and Functional Characterization of Distinct Breg Subsets in Uninfected and Infected Mice. (A) UMAP plot for B cell subtypes. (B) Violin plot for the MDSC signature in B cell subtypes. (C) Dot plot showing the expression of IL‐10 in B cell subtypes. (D) Comparison of IL‐10 expression in 3 types of Breg between uninfected and infected groups. (E) Density plot reveals that Cd274, Ahr, and Tim1 exhibit high levels of expression in 3 distinct types of Bregs, respectively. (F,G) FC analysis of Bregs, PD‐L1^+^ Bregs, Ahr^+^ Bregs, and Tim1^+^ Bregs between uninfected and infected groups (n = 5 per groups). (H,I) Flow cytometry analysis of the effects of different Breg subgroups on GZMB secretion in activated T cells (n = 5 per groups). (Bar plot displays the means ±SD; ***p < 0.001).

The suppression of T cells by PMN‐MDSCs is a well‐defined immune escape mechanism. However, our analysis revealed that CXCR4^+^ PMN‐MDSCs exerted the most significant signal regulation on B cells, suggesting the potential involvement of specific B cell subtypes in promoting disease progression in PJI. To investigate this further, we conducted secondary dimensionality reduction clustering on B cells and categorized them into 10 subgroups based on classical biomarkers. These included 4 naive B cell subgroups, 2 memory B cell subgroups, 2 germinal center B cell subgroups, pre‐B cells, and pre‐GC cells, among which the proportions of Bn_stressed, Bm_Bank1^+^, pre‐GC, and B_GC_Rgs13^+^ were increased in the SA group (Figure 3A, Figure S7A,B). Bregs, a widely studied subset of immunosuppressive B cells in recent years, facilitate immune escape through intercellular contact and the secretion of various factors. According to the Breg signature evaluation, we observed significantly higher scores in Bm_Ets1^+^, pre‐GC, and BGC_Rgs13^+^ subgroups compared to others (Figure 3B). To substantiate our findings, we evaluated the expression of IL‐10, a core secretory factor of Bregs. Consistent with our hypothesis, IL‐10 exhibited the highest expression levels in the aforementioned three subgroups (Figure 3C). Notably, IL‐10 expression was significantly elevated in the infected group compared to the control group across these three B cell subgroups, underscoring the importance of IL‐10 secreted by Bregs in PJI (Figure 3D). Each of the three Breg subgroups displayed distinct characteristics. Bm_Ets1^+^ highly expressed Cd274 (PD‐L1), pre‐GC highly expressed Ahr, and B_GC_Rgs13^+^ highly expressed Tim1, all of which are recognized as classic Breg marker genes (Figure 3E). Functionally, Bm_Ets1^+^ primarily plays a primary role in regulating inflammation and energy metabolism, while pre‐GC simultaneously inhibits T cell and B cell functions. B_GC_Rgs13^+^, on the other hand, focuses on inhibiting T cell activation and antigen presentation processes (Figure S7C–E). Furthermore, FC analysis confirmed the high abundance of Bregs in PJI mice and revealed a significant increase in the proportion of the three Breg subtypes during infection (Figure 3F,G). Intriguingly, deconvolution‐based estimation of immune cell proportions in human PJI datasets revealed that the relative abundances of all three Breg subtypes were consistently elevated in the PJI cohort compared to controls (Figure S7F). This clinical observation aligns with our experimental findings and underscores the potential relevance of these Breg subpopulations in the pathogenesis of PJI.

T cell suppression is a hallmark characteristic of Bregs. To determine whether the newly identified Breg subsets share this functional property, we isolated the three Breg subpopulations from the periarticular soft tissues of PJI mice via FACS, based on their specific surface markers, and subsequently co‐cultured them with T cells (Figure S7G). Our results demonstrated that all three subsets exerted inhibitory effects on the secretion of antimicrobial effectors by T cells to varying extents (Figure 3H,I, Figure S7H,I). Collectively, our findings indicate that Bregs with diverse functions are present in PJI and may play an unexpected role in immune evasion.

**FIGURE 4:**
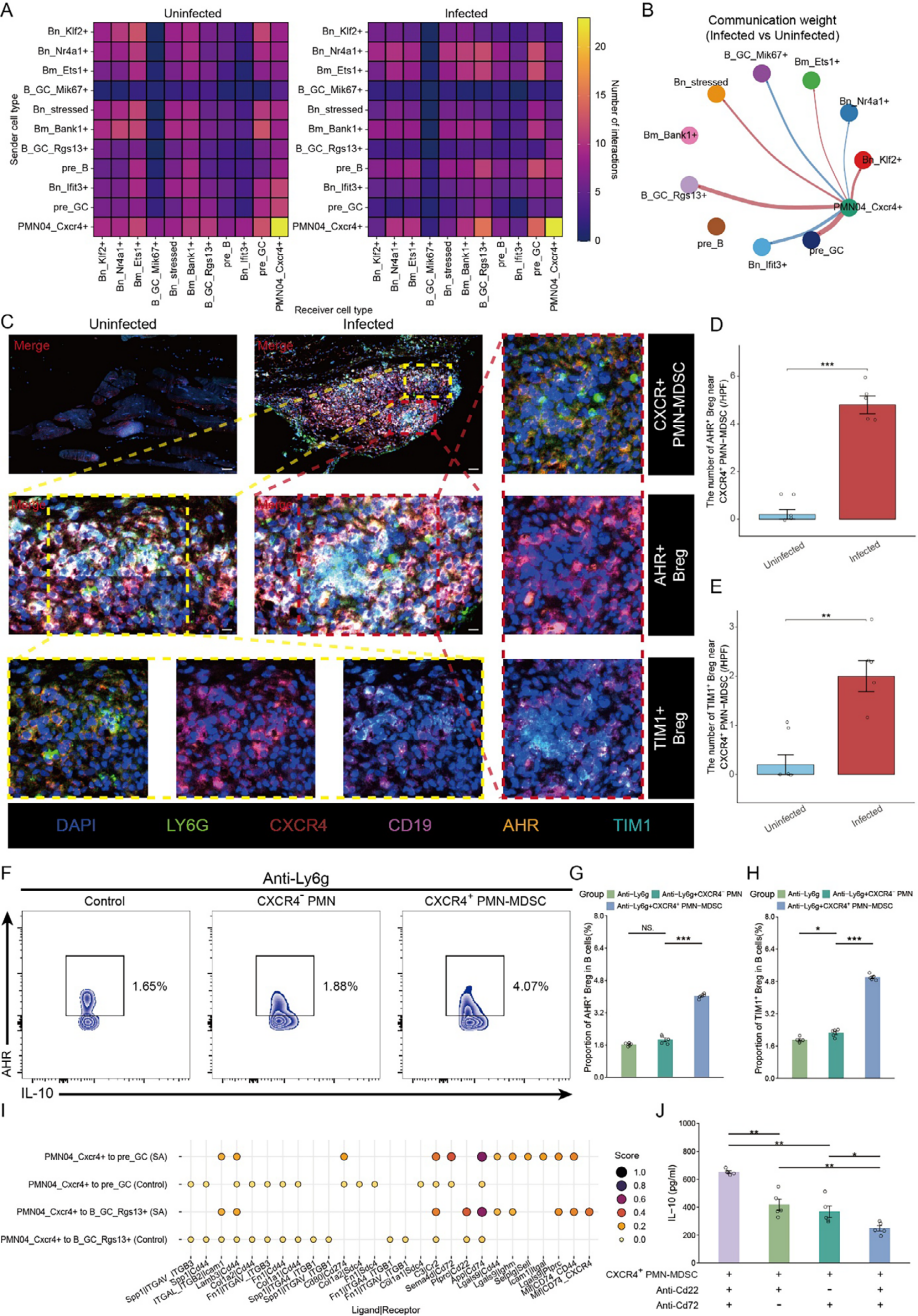
CXCR4^+^ PMN‐MDSCs Interact with Bregs to Form an Immunosuppressive Network in PJI. (A) A heatmap presenting the cell‐cell communication number between CXCR4^+^ PMN‐MDSCs and B cell subtypes in both uninfected and infected groups. (B) A circle network presenting the cell‐cell communication weight between CXCR4^+^ PMN‐MDSCs and B cell subtypes in both uninfected and infected groups. (C–E) Multiplex immunofluorescence image (including merge and single figures) and statistics analysis spatially demonstrate the interaction between CXCR4^+^ PMN‐MDSCs and Breg subtypes (n = 5 per group, scale bar: 50 µm for LPF and 10 µm for HPF). (F) Flow cytometry displays the effects of depleted PMN‐MDSCs and adoptive transfer of CXCR4^−^ PMNs or CXCR4^+^ PMN‐MDSCs on AHR^+^ Bregs. (G,H) Statistical analysis of the effects of adoptive transfer of CXCR4^−^ PMNs or CXCR4^+^ PMN‐MDSCs on the proportion of different Breg subtypes (n = 5 per group). (I) Bubble plot showing ligand‐receptor interactions of CXCR4^+^ PMN‐MDSC with pre‐GC and Rgs13^+^ GC cells, respectively. (J) ELISA detection of IL‐10 secretion in B cells co cultured with CXCR4^+^ PMN‐MDSCs using anti‐CD22 (10 µg/mL) and anti‐CD72 (10 µg/mL) alone or in combination (n = 5 per group). (Bar plot displays the means ±SD; ns = not significant, * *p* < 0.05, **p < 0.01, ***p < 0.001).

Communication analysis revealed that CXCR4^+^ PMN‐MDSCs exhibited significantly increased outgoing signals toward pre‐GC and B_GC_Rgs13^+^ Breg subtypes in the infected group (Figure 4A,B). This finding suggested that CXCR4^+^ PMN‐MDSCs exert more pronounced regulatory effects on these two Breg subtypes. The interaction between them was further substantiated by multiple immunofluorescence staining. During PJI infection, there was a marked increase in Arh^+^ Bregs and Tim1^+^ Bregs around CXCR4^+^ PMN‐MDSCs (Figure 4C–E). Furthermore, we sought to validate the aforementioned cellular interactions in vivo. Initially, PMN‐MDSCs were depleted using an anti‐Ly6g neutralizing antibody, with the depletion efficiency confirmed by flow cytometric analysis (Figure S8A,B). Subsequently, either CXCR4^−^ PMNs or CXCR4^+^ PMN‐MDSCs were adoptively transferred into the depleted mice. Notably, the administration of CXCR4^+^ PMN‐MDSCs, but not CXCR4^−^ PMNs, significantly increased the proportions of AHR^+^ and TIM1^+^ Bregs (Figure 4F–H, Figure S8C,D). These findings demonstrate that CXCR4^+^ PMN‐MDSCs specifically—rather than other PMN subsets—interact with these characteristic Breg subpopulations in vivo.

Ligand‐receptor analysis suggested potential differences in the interaction of CXCR4^+^ PMN‐MDSCs with AHR^+^ and TIM1^+^ Bregs. The Ptprc‐Cd22 axis appeared more prominent in TIM1^+^ Breg interactions, while Sema4d‐Cd72 seemed to contribute more to the AHR^+^ subset (Figure 4I). Consistently, within the co‐culture system of B cells and CXCR4^+^ PMN‐MDSCs, neutralizing CD22 or CD72 individually led to a partial reduction in IL‐10 secretion, with a further decrease observed upon dual blockade (Figure 4J). These preliminary findings suggest that these pathways may, at least in part, mediate the interaction between CXCR4^+^ PMN‐MDSCs and Bregs. In summary, the regulation of Bregs by CXCR4^+^ PMN‐MDSCs form a crucial component of the immune suppression network in PJI, offering a novel perspective for alleviating immune suppression in infectious contexts.

### 2.5 Inhibition of CXCR4 Expression can Decreases Breg Abundance and Immunosuppressive Factor Production

**FIGURE 5:**
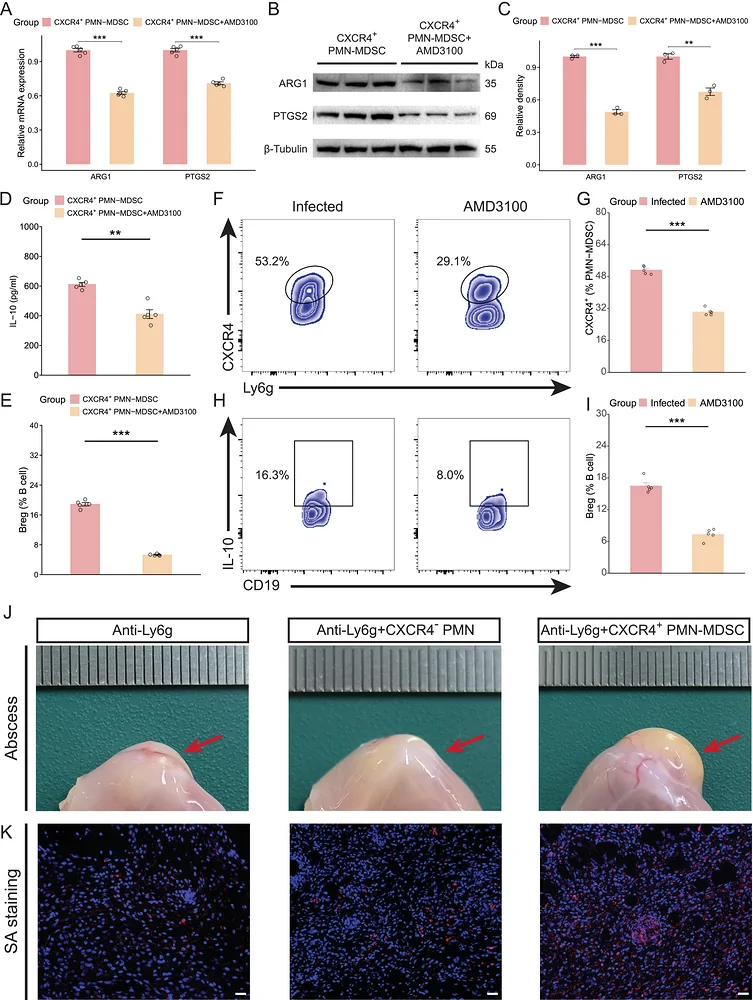
CXCR4^+^ PMN‐MDSCs Promote Immunosuppression and Exacerbate PJI via the CXCR4 Axis. (A) qRT‐PCR of ARG1 and PTGS2 in CXCR4^+^ PMN‐MDSCs with or without AMD3100 (n = 5 per group). (B,C) WB of ARG1 and PTGS2 in CXCR4^+^ PMN‐MDSCs with or without AMD3100 (n = 3 per group). (D) ELISA measurement of IL‐10 secretion from B cells co‐cultured with CXCR4^+^ PMN‐MDSCs with or without AMD3100 (n = 5 per group). (E) Flow cytometry analysis of Breg proportion from B cells co‐cultured with CXCR4^+^ PMN‐MDSCs with or without AMD3100 (n = 5 per group). (F‐I) Flow cytometry analysis of CXCR4^+^ PMN‐MDSCs, Bregs, GZMB and IFNG in CD8^+^ T cells in the knee joint soft tissue of PJI mice treated with or without AMD3100 (n = 5 per groups). (J) Gross view of the knee joint in the following three groups: anti‐Ly6g treatment, adoptive transfer of CXCR4^−^ PMNs, and adoptive transfer of CXCR4^+^ PMN‐MDSCs. (K) Representative immunofluorescence images of anti‐SA antibody in the indicated groups (scale bar: 20 µm). (Bar plot displays the means ±SD; ** *p* < 0.01, ***p < 0.001.).

Because the high abundance of CXCR4^+^ PMN‐MDSCs can recruit Bregs and thereby exacerbate PJI, we explored whether inhibiting CXCR4 could mitigate this effect. Treatment with AMD3100, a well‐known selective CXCR4 antagonist [32], appeared to downregulate the expression of ARG1 and PTGS2 in CXCR4^+^ PMN‐MDSCs at both the mRNA and protein levels in vitro (Figure 5A–C). Consistently, CXCR4 inhibition in these cells was associated with reduced IL‐10 secretion in co‐cultured B cells (Figure 5D,E). These observations suggest that CXCR4 signaling may play a role in the induction of Breg cells by CXCR4^+^ PMN‐MDSCs.

Given that CXCR4 can be expressed across various cell types, we first examined its expression profile using scRNA‐seq data from PJI mice. The results indicated that neutrophils exhibited the highest frequency of CXCR4 expression among the identified populations (Figure S9A,B). Based on these observations, we utilized AMD3100 as a preliminary pharmacological intervention to explore the potential role of the CXCR4 axis in our PJI model. Following AMD3100 treatment in PJI mice, the percentages of CXCR4^+^ PMN‐MDSCs and Bregs decreased significantly. (Figure 5F–I). Then, following the aforementioned depletion and adoptive transfer protocol, we observed that PMN‐MDSC deficiency led to smaller abscesses and lower bacterial loads. Notably, re‐introducing CXCR4^+^ PMN‐MDSCs, rather than CXCR4^−^ PMNs, effectively recapitulated the infected phenotype, evidenced by enlarged abscesses and increased bacterial loads. These results indicate that CXCR4^+^ PMN‐MDSCs may serve as a key subset driving the exacerbation of infection. (Figure 5J,K).

### 2.6 Alendronate Inhibits the Function of CXCR4^+^ PMN‐MDSCs by Targeting STAT3

**FIGURE 6:**
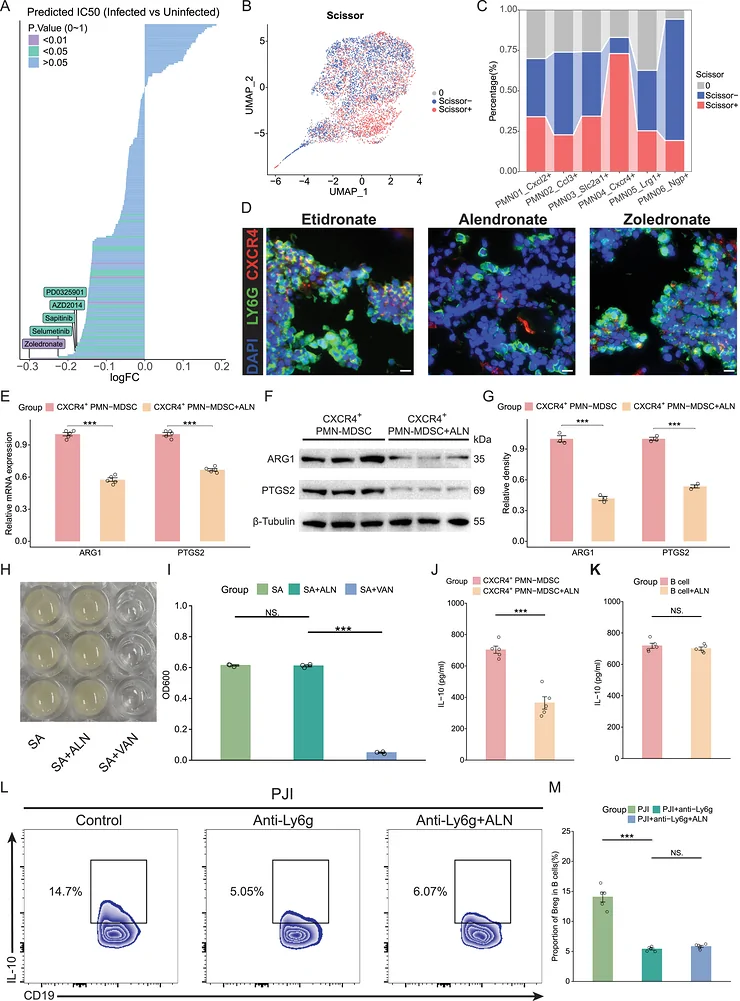
Alendronate Blocks the Immunosuppressive Network by Regulating CXCR4^+^ PMN‐MDSCs. (A) Comparison of IC50 differences between groups, identifying sensitive drugs for CXCR4^+^ PMN‐MDSCs in the infection group. IC50 results calculated using the oncoPrectict algorithm. (B,C) The Scissor algorithm validate PMN subtypes' sensitivity to alendronate, and Scissor^+^ cells are indicative of a heightened sensitivity to alendronate. (D) Representative immunofluorescence images of CXCR4^+^ PMN‐MDSCs in knee joint soft tissue of mice treated with etidronate, alendronate, and alendronate (scale bar: 10 µm). (E) qRT‐PCR of ARG1 and PTGS2 in CXCR4^+^ PMN‐MDSCs with or without alendronate (n = 5 per group). (F,‐G) WB of ARG1 and PTGS2 in CXCR4^+^ PMN‐MDSCs with or without alendronate (n = 3 per group). (H,I) Evaluation of the direct antimicrobial activity of alendronate via OD600 spectrophotometry, with vancomycin serving as a positive control. (J) IL‐10 levels in B cells co‐cultured with alendronate‐pretreated CXCR4^+^ PMN‐MDSCs. (K) ELISA measurement of IL‐10 secretion from B cells treated with or without alendronate before co‐culture with CXCR4^+^ PMN‐MDSCs. (L,M) FC plots and statistics plot of Bregs in PJI mice treated with anti‐Ly6g alone or in combination with alendronate (n = 5 per groups). (Bar plot displays the means ±SD; ns = not significant, ***p < 0.001).

AMD3100 is a widely employed CXCR4 inhibitor; however, cells with high CXCR4 expression are not exclusive to CXCR4^+^ PMN‐MDSCs. Consequently, we cannot definitively determine whether AMD3100 will cause side effects by targeting other CXCR4‐expressing cells. Identifying specific targets for CXCR4^+^ PMN‐MDSC holds significant clinical value. To achieve this, we first estimated the IC50 values of PMN cell subtypes using the oncoPredict package and selected zoledronate as a potential CXCR4^+^ PMN‐MDSC‐sensitive drug based on the disparity in IC50 between infected and non‐infected groups (Figure 6A). The Scissor algorithm further mapped zoledronate's drug sensitivity information to scRNA data, revealing that CXCR4^+^ PMN‐MDSCs were the most effectively targeted cell type by zoledronate, with a sensitive cell proportion of 73.01% (Figure 6B,C). Zoledronate is a third‐generation bisphosphonate. To evaluate the targeting efficacy of bisphosphonates on CXCR4^+^ PMN‐MDSC, first‐generation etidronate, second‐generation alendronate, and third‐generation zoledronate were injected into PJI mice. The results indicated that alendronate appeared to exert a superior inhibitory effect on CXCR4^+^ PMN‐MDSCs (Figure 6D). Furthermore, at appropriate drug concentrations, etidronate and alendronate did not significantly impact cell viability, whereas zoledronate markedly increased the apoptosis rate of MC3T3‐E1 cells from a concentration of 10 mM (Figure S10A). Ultimately, we selected alendronate as a potential drug targeting CXCR4^+^ PMN‐MDSCs for the treatment of PJI.

To evaluate the effects of alendronate, we first treated CXCR4^+^ PMN‐MDSCs with alendronate in vitro. Consistent with our hypothesis, alendronate treatment reduced the expression of ARG1 and PTGS2 at both the mRNA and protein levels (Figure 6E–G). Next, we examined whether alendronate possesses direct antibacterial activity. Interestingly, alendronate failed to significantly inhibit S. aureus growth after overnight incubation, suggesting that its therapeutic effect is not mediated by direct bactericidal action (Figure 6H,I). To further pinpoint its cellular target, we performed crossover co‐culture assays. Alendronate pre‐treatment of CXCR4^+^ PMN‐MDSCs markedly diminished IL‐10 secretion in co‐cultured B cells; however, this effect was abolished when only B cells were pre‐treated with alendronate, indicating that alendronate acts primarily on CXCR4^+^ PMN‐MDSCs rather than B cells (Figure 6J,K). This target specificity was further substantiated in vivo, as alendronate administration failed to further reduce Breg proportions in PMN‐MDSC‐depleted mice. (Figure 6L,M) Collectively, these results suggest that the inhibitory effect of alendronate on Breg expansion is likely dependent on its direct action on CXCR4^+^ PMN‐MDSCs.

**FIGURE 7:**
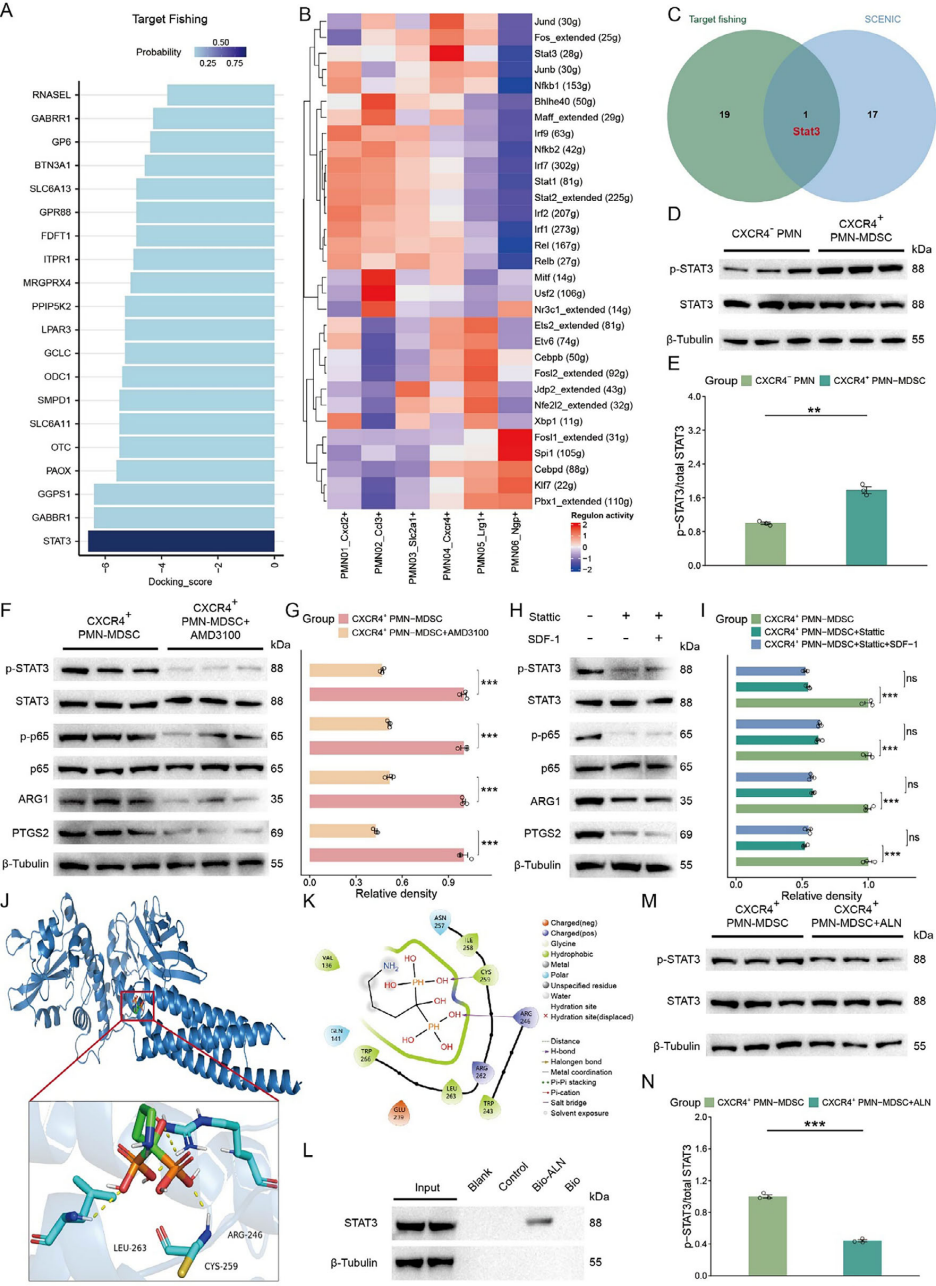
Alendronate Inhibits the Function of CXCR4^+^ PMN‐MDSCs by Targeting STAT3. (A) Target fishing for the top 20 potential proteins targeted by alendronate. (B) SCENIC analysis to identify key TF across PMN subtypes. (C) Venn diagram to identifying the intersection of CXCR4+ PMN‐MDSC hub TFs and potential targets of alendronate. (D,E) WB analysis of p‐STAT3 between CXCR4^−^ PMNs and CXCR4^+^ PMN‐MDSCs. (F–I) WB analysis of p‐STAT3 and downstream molecules in CXCR4^+^ PMN‐MDSCs following treatment with the CXCR4 antagonist AMD3100 (F,G), or the STAT3 inhibitor Stattic (10 µM) with/without the CXCR4 agonist SDF‐1 (H,I) (n = 3 per groups). (J,K) Molecular docking between alendronate and STAT3, and the enlarged image shows the interaction forces. (L) Streptavidin pulldown from CXCR4^+^ PMN‐MDSCs lysates probed by immunoblot for STAT3. Blank (beads only), Ctrl (pulldown control), Input (whole cell lysate), Bio‐Cel (pulldown with biotinylated alendronate), Biotin (biotin control). (M,N) WB analysis of p‐STAT3 in CXCR4^+^ PMN‐MDSCs following treatment with alendronate (n = 3 per groups). (Bar plot displays the means ±SD; ns = not significant, ** *p* < 0.01, ***p < 0.001.).

Next, target fishing was employed to identify alendronate's drug targets. Based on the docking score and probability of binding, STAT3 emerged as the optimal binding protein, with a docking score of −6.6 kcal/mol (Figure 7A). To identify the central therapeutic target of alendronate, we reasoned that such a target should ideally represent a core TF within CXCR4^+^ PMN‐MDSCs. Utilizing the SCENIC algorithm to profile TF regulatory networks across PMN subpopulations (Figure 7B), we intersected these candidates with potential alendronate targets identified via inverse docking analysis. The Venn diagram highlighted STAT3 as a promising intersection (Figure 7C). Experimental validation revealed that CXCR4^+^ PMN‐MDSCs exhibited significantly higher levels of STAT3 phosphorylation compared to CXCR4^−^ PMNs, suggesting that STAT3 activation is a defining feature of this subpopulation (Figure 7D,E). Furthermore, AMD3100 treatment blunted STAT3 phosphorylation and its downstream effectors, including p65 phosphorylation and immunosuppressive molecules such as ARG1 (Figure 7F,G). Notably, the regulatory role of CXCR4 in PMN‐MDSC function appeared to be STAT3‐dependent; pharmacological inhibition of STAT3 using stattic effectively abolished the restorative effects of CXCR4 agonists on STAT3 phosphorylation and downstream gene expression (Figure 7H,I). These results suggest that the CXCR4‐STAT3 axis may serve as a critical regulatory pathway in CXCR4^+^ PMN‐MDSCs.

Molecular docking results elucidated the interaction between alendronate and STAT3, revealing that the ARG246 and CYS259 residues on the STAT3 receptor formed hydrogen bonds with alendronate. Additionally, the GLU239, TRP266, LEU263, ARG262, TRP243, VAL136, ILE258, and ASN257 residues also formed van der Waals interactions with alendronate (Figure 7J,K). Furthermore, molecular dynamics simulations were used to understand the motion trajectory and conformation of their binding. The Rg results showed that the complex fluctuated relatively steadily during movement, indicating that the small molecule target protein complex did not undergo significant expansion or contraction during movement (Figure S10B). The SASA results indicate that ligand binding has little effect on protein structure (Figure S10C). The RMSF of the STAT3‐alendronate complex was relatively low, indicating weak flexibility but stable structure (Figure S10D). To substantiate the possibility of in vivo binding between the two molecules, a pull‐down experiment was performed using bio‐alendronate, and consistently, the binding of bio‐alendronate to STAT3 was observed in the cell lysate of CXCR4^+^ PMN‐MDSCs (Figure 7L). Furthermore, alendronate significantly inhibited STAT3 phosphorylation in CXCR4^+^ PMN‐MDSCs in vitro (Figure 7M,N). Intriguingly, colivelin treatment recapitulated the stimulatory effect of CXCR4^+^ PMN‐MDSC on B cell IL‐10 production despite alendronate pretreatment, confirming that STAT3 is the functional target of alendronate (Figure S10E). Notably, while STAT3 is also a known regulator of B cell function, alendronate treatment did not markedly alter STAT3 phosphorylation levels in B cells themselves (Figure S10F,G). This observation implies that alendronate may possess a relatively high degree of selectivity for CXCR4^+^ PMN‐MDSCs over B cells.

### 2.7 Alendronate Combined With Vancomycin Attenuates Skin Infection by Reversing Immunosuppression

**FIGURE 8:**
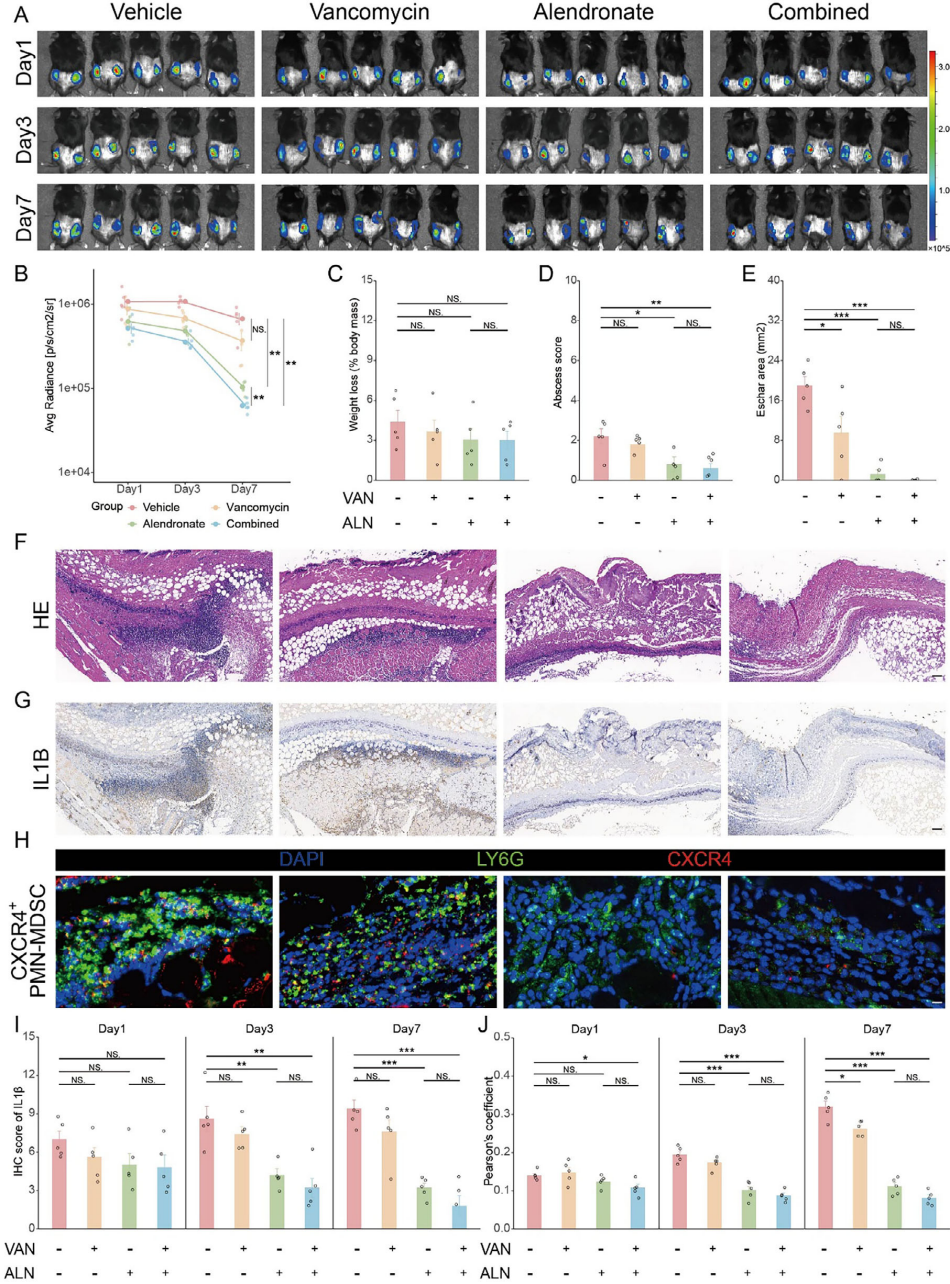
Alendronate Combined with Vancomycin Attenuates Skin Infection by Reversing Immunosuppression. (A,B) Utilize a live imaging system to conduct assessment of the infection burden in mice with skin infection at various time intervals following treatment with PBS, vancomycin, alendronate, or both together. The average radiance level serves as an index to determine the content of S. aureus. (C–E) Statistical analysis to evaluate the weight loss, abscess score, and eschar area in mice with skin infection across different groups on day 7. (F) H&E staining reveals the morphological characteristics of the inflammatory region in skin infections of day 7 in the indicated groups (scale bar: 100 µm). (G) Immunohistochemical staining of IL‐1β in skin infections of day 7 in the indicated groups (scale bar: 100 µm). (H) Representative immunofluorescence images of CXCR4^+^ PMN‐MDSCs in skin infections of day 7 in the indicated groups (scale bar: 10 µm). (I,J) Statistics analysis of IHC score of IL‐1β and Pearson coefficient of CXCR4^+^ PMN‐MDSCs (n = 5 per groups). (Bar plot displays the means ±SD; ns = not significant, *p < 0.05, **p < 0.01, ***p < 0.001.).

It is noteworthy to explore whether the inhibition of CXCR4^+^ PMN‐MDSCs by alendronate facilitates the recovery from bacterial infections. Before that, we initially conducted an assessment of alendronate's drug safety. Across a concentration range spanning from 0.125 to 128 µg/mL, alendronate alone or in combination with vancomycin exhibited no significant hemolytic activity (Figure S11A,B). Meanwhile, alendronate treatment, alone or combined with vancomycin, caused no significant hepatotoxicity or systemic inflammation. Histopathological analysis further confirmed the absence of structural damage in vital organs, indicating that these therapeutic interventions were well‐tolerated without apparent side effects. (Figure S11C–G). After confirming alendronate's safety, we continuously monitored the bacterial load in skin infections on days 1, 3, and 7 using the Xen29 strain. The results revealed that both alendronate treatment alone (ALN group) and its combination with vancomycin (Comb group) significantly reduced bacterial counts by day 7, with the combined therapy proving more effective (Figure 8A,B). Following alendronate treatment, the abscess score in the infected skin of mice decreased markedly, as did the area of eschar, indicating that alendronate exerts a certain effect in alleviating skin infections, particularly when used in conjunction with vancomycin (Figure 8C–E).

Regarding inflammation, no significant anti‐inflammatory effect was observed in the ALN or Comb groups on day 1. However, on day 3 and 7, the inflammatory infiltration area of the skin in these two groups decreased substantially, and the expression of IL‐1β also decreased notably, suggesting that alendronate plays a role in partially relieving inflammation (Figure 8F,G,I; Figure S12A,B). Simultaneously, from the first day of infection, both the ALN group and Comb group significantly reduced the proportion of CXCR4^+^ PMN‐MDSCs, confirming the targeting effect of alendronate on these cells (Figure 8H,J; Figure S12C).

### 2.8 Combined Alendronate and Vancomycin Reduces Bacterial Burden, Enhances Antibacterial Immune Responses, and Attenuates Osteolysis in PJI

**FIGURE 9:**
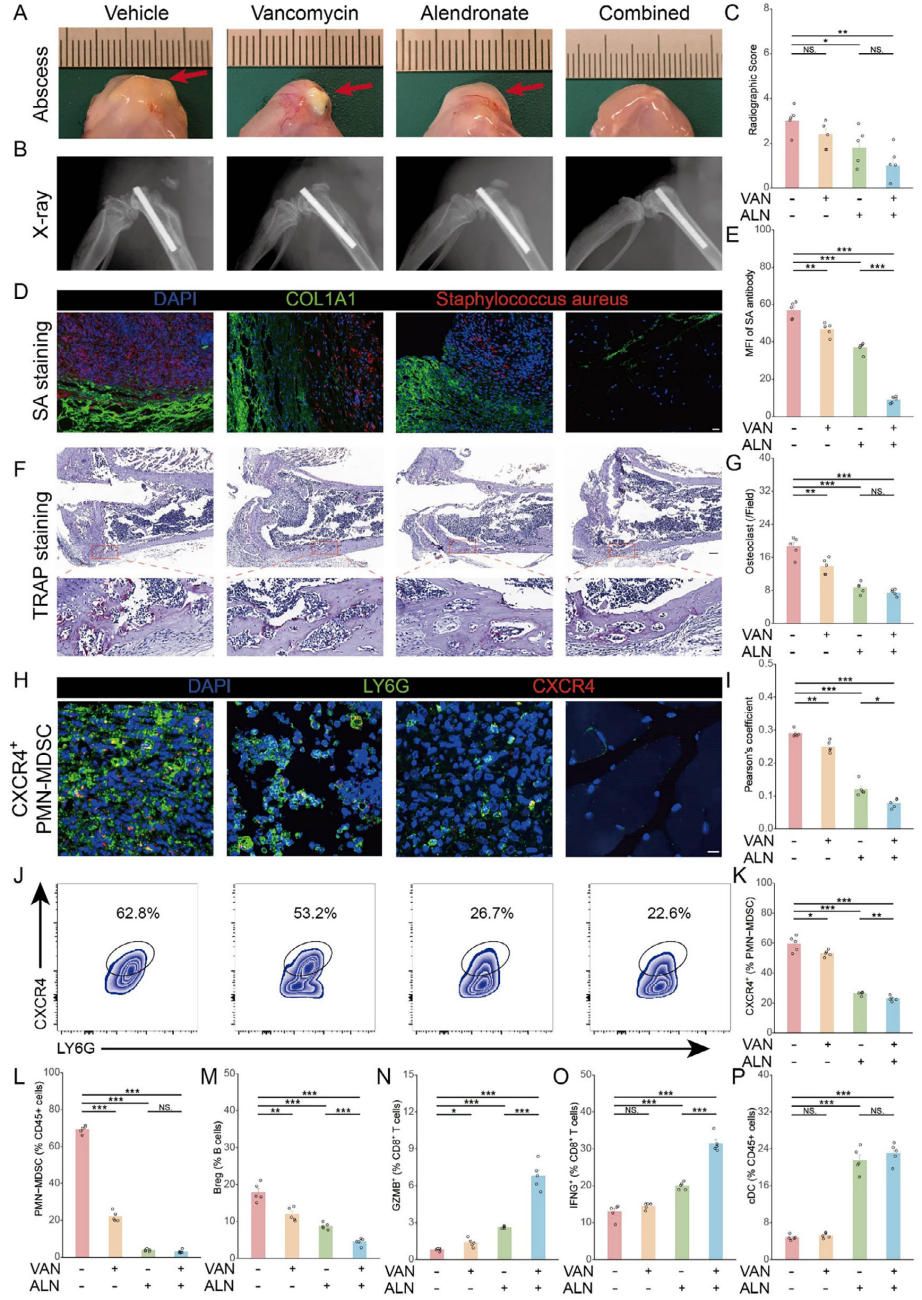
Combined Alendronate and Vancomycin Reduces Bacterial Burden, Enhances Antibacterial Immune Responses, and Attenuates Osteolysis in PJI. (A) Gross view of the knee joint in mice with PJI following treatment with PBS, vancomycin, alendronate, or both together. (B,C) Radiographic scores in the indicated groups (n = 5 per groups). (D,E) Representative immunofluorescence images of COL1A1 and anti‐SA antibody in the indicated groups (n = 5 per groups, scale bar: 10 µm). (F,G) Trap staining was employed to conduct a quantitative assessment of osteoclast numbers within the femurs of PJI mice bearing implants (n = 5 per groups, scale bar: 200 µm for LPF and 20 µm for HPF). (H,I) Representative immunofluorescence images of CXCR4^+^ PMN‐MDSCs in the soft tissues surrounding the knee joint in the indicated groups (scale bar: 10 µm). (J–P) FC analysis of CXCR4^+^ PMN‐MDSCs, Bregs, GZMB and IFNG in CD8+ T cells, cDCs, and PMN‐MDSCs in the indicated groups (n = 5 per groups). (Bar plot displays the means ±SD; ns = not significant, *p < 0.05, **p < 0.01, ***p < 0.001.).

CXCR4^+^ PMN‐MDSCs and Bregs are critical components of the immune suppression network in PJI, suggesting that alendronate, which targets CXCR4^+^ PMN‐MDSCs, may serve as an effective therapeutic agent for alleviating immune suppression in PJI. Following treatment with alendronate, a significant shrinkage of abscess area around the knee joint was observed, and X‐ray imaging further revealed that alendronate mitigated bone destruction in the femur (Figure 9A–C). Notably, combination therapy with vancomycin nearly healed the infection. Both the ALN group and the Comb group significantly reduced Staphylococcus aureus count and facilitated a more orderly collagen deposition (Figure 9D,E).

Bone resorption and destruction are distinctive features of PJI compared to other infections. Owing to alendronate's inherent anti‐osteoporotic effects, the number of osteoclasts decreased markedly after alendronate treatment (Figure 9F,G). Concurrently, the ALN group and Comb groups demonstrated promising outcomes in bone reconstruction and healing. Bone mineral density, bone volume/total volume, and trabecular thickness all increased to varying degrees, reflecting enhanced bone mineralization and increased bone mass. Additionally, trabecular separation and the structure model index decreased significantly, indicating a more compact bone structure (Figure S13A). Notably, even after the depletion of PMN‐MDSCs, alendronate monotherapy was still able to partially rescue bone mass and remodel bone architecture. These observations suggest that alendronate may possess an intrinsic bone‐repairing effect that is independent of its role in modulating the osteoimmune microenvironment (Figure S13B). Alendronate treatment even alleviated inflammation levels in bone tissue and bone marrow, as evidenced by the downregulation of IL‐1β and TNF‐α expression (Figure S14A–E). In summary, both alendronate monotherapy and its combination with vancomycin significantly reduced the soft tissue infection burden and enhanced bone repair and reconstruction in PJI.

Antibacterial immunity relies on the harmonious coordination of the immune microenvironment. Immunofluorescence analysis revealed that treatment with alendronate alone or in combination with antibiotics significantly reduced the abundance of CXCR4^+^ PMN‐MDSCs within abscess lesions (Figure 9H,I). Based on the results of flow cytometry, alendronate treatment significantly reduced the proportion of CXCR4^+^ PMN‐MDSCs and total PMN‐MDSCs (Figure 9J–L; Figure S15A). Concurrently, there was a marked decrease in the number of Bregs (Figure 9M; Figure S15B). Conversely, CD8^+^ T cells, which play a pivotal role in adaptive immunity, secreted increased levels of antibacterial substances, including GZMB and IFNG, while the proportion of cDCs involved in antigen presentation also increased significantly (Figure 9N–P; Figure S15C–E). The reduction of immunosuppressive cells and the augmentation of antibacterial immune cells collectively dismantled the immunosuppressive microenvironment of PJI, establishing favorable tissue homeostasis for PJI recovery.

## 3 Discussion

At present, there's a lack of in‐depth analysis regarding the immunosuppressive network in bacterial infections, especially PJI. While a handful of recent studies have centered on the role of MDSCs in facilitating biofilm persistence in PJI [8, 33], there has been neither subtyping of MDSCs nor elucidation of their relationship with the immune suppression network. In this study, we comprehensively analyzed the heterogeneity of PMN‐MDSCs in PJI using scRNA sequencing. We discovered that a distinct subset of CXCR4^+^ PMN‐MDSCs exerts a pivotal immunosuppressive function during infection, characterized by the expression of inhibitory molecules and modulation of Bregs. Although PMN‐MDSCs and their interactions with Bregs have been reported in cancer research—where they primarily mediate tumor immune evasion and metastasis—their role in infectious diseases remains largely unexplored [34]. To our knowledge, this is the first study to identify this cellular crosstalk within the unique immunosuppressive microenvironment of PJI, thereby establishing a novel immunoregulatory function for this specific CXCR4^+^ subpopulation. The drug screening strategy targeting CXCR4^+^ PMN‐MDSCs identified alendronate as an effective drug, mainly by binding to the key transcription factor STAT3. To enhance therapeutic efficacy, the combination of alendronate and vancomycin has yielded promising results in skin infection and PJI models, including abscess resolution, inflammation alleviation, and enhanced bone regeneration and repair. Most importantly, the combination therapy effectively disrupts the immunosuppressive network, as evidenced by reduced PMN‐MDSCs and Bregs, alongside increased CD8^+^ T cells secreting antibacterial factors and antigen‐presenting cDCs. CXCR4^+^ PMN‐MDSC, as an immune suppressive cell widely found in bacterial infections, is expected to become a new diagnostic and therapeutic target for bacterial infections

Since its discovery, MDSCs has been a focal point of research. However, owing to its pronounced heterogeneity, MDSCs frequently displays distinct transcriptional features across different tumors. Simultaneously, it exhibits a high degree of functional diversity and acute sensitivity to the microenvironment, resulting in a one‐sided understanding of them [35]. MDSCs demonstrate significant heterogeneity not only in tumors but also in bacterial infections. In our study, we found that in addition to M‐MDSCs, there were two subtypes of PMN‐MDSCs in PJI, which highly expressed Slc2a1 and Cxcr4, respectively. Slc2a1 encodes a glucose transporter protein involved in glycolysis, facilitating tumor metastasis, which aligns with the upregulation of glycolysis in PMN‐MDSCs [8, 36]. The immunosuppressive effect of PMN04_Cxcr4^+^ on immune cells is stronger than that of PMN03_Slc2a1^+^, so we focus on CXCR4^+^ PMN‐MDSCs. CXCR4 is a G protein‐coupled receptor that regulates cell proliferation, apoptosis, autophagy, and other processes to varying extents [32]. Neutrophils with high CXCR4 expression have been observed in various diseases, yet their roles and characteristics vary. In tumors, CXCR4^+^ neutrophils are marked by promoting angiogenesis and exerting strong immunosuppression, indicating a poor tumor prognosis [37, 38]. In sepsis and viral infections, CXCR4^+^ neutrophils often appear during bone marrow emergency hematopoiesis, triggering robust inflammation and tissue damage [39, 40]. While CXCR4^+^ neutrophils in autoimmune diseases have been reported to overexpress pro‐inflammatory factors and exhibit enhanced phagocytic and degranulation capabilities [41], our findings suggest that these cells can adopt distinct functional states in response to diverse pathological signals. In this study, CXCR4^+^ neutrophils were identified for the first time in PJI infection, displaying transcriptional and differentiation characteristics of PMN‐MDSCs. They exhibit a dual nature of pronounced inflammatory activation and downregulated bactericidal function against Staphylococcus aureus infection, differing from previously identified neutrophils with high CXCR4 expression. It is noteworthy that numerous studies have linked a type of neutrophil with high CXCR4 expression to aging and inflammation. These neutrophils also exhibit elevated ARG1 levels and poor antibacterial activity [42, 43]. However, the CXCR4^+^ PMN‐MDSCs identified in this study is characterized by high expression of classic MDSC markers such as IL1B and ARG1, rather than upregulation of aging‐related genes like p16 and p21. Furthermore, co‐culturing FACS‐isolated CXCR4^+^ neutrophils with T cells revealed a significant suppression of T cell proliferative and secretory capacities. These observations suggest that CXCR4^+^ neutrophils may represent a specialized subpopulation with potent immunosuppressive properties. In conclusion, although we have designated the CXCR4^+^ PMN‐MDSCs as a novel subpopulation in this study, these cells might instead represent a specific functional state of neutrophils rather than a terminal lineage. Further granular functional validation is warranted in future investigations to more precisely define their identity.

Intercellular communication often serves as a key mechanism for cellular function. In this study, Bregs were identified as the cell type with the strongest interaction with CXCR4^+^ PMN‐MDSCs. Initially, research on Bregs primarily focused on autoimmune diseases such as rheumatoid arthritis, where Bregs secrete anti‐inflammatory factors like IL‐10 and TGF‐β to inhibit the proliferation of pathogenic T cells and pro‐inflammatory cells, while promoting the differentiation of Tregs [44, 45]. This synergistic regulation of immune responses helps alleviate disease progression. However, in infectious diseases, the role of Bregs varies significantly depending on the infection state. Under conditions of excessive inflammation, Breg can limit the inflammatory response and enhance the antibacterial effect in mice [46]. In most cases, particularly in chronic infections, Bregs may be exploited by pathogens, inhibiting the bactericidal function of immune cells such as T cells (including Th1, Th17, etc.) and macrophages, thereby promoting infection deterioration [47, 48, 49]. In summary, it is essential to consider the role of Bregs in the immunosuppressive microenvironment. Our initial analysis revealed that B cells constitute a substantial proportion of the immune cell infiltrate in PJI, second only to neutrophils. This prominent accumulation prompted us to investigate the potential functional roles of B cells in this context. Given the established immunosuppressive capacity of Bregs, we reasoned that a subset of these B cells might possess a Breg phenotype and contribute to the immune landscape of PJI. Interestingly, we found that Breg not only exists in PJI but can also be divided into three distinct subtypes, indicating heterogeneity of Breg in PJI. Due to limited research on Bregs in bacterial infections, we have temporarily named these subtypes using reported Breg markers rather than introducing new markers to facilitate a better understanding of these Breg subtypes [50]. In the future, we plan to conduct more detailed research on Breg in PJI. In this study, it was observed that Bregs with high expression of AHR and TIM1 is the primary target of CXCR4^+^ PMN‐MDSCs. AHR is a crucial transcription factor for B cell differentiation, and its high expression can inhibit the pro‐inflammatory differentiation of B cells [51]. TIM1 has been reported to be induced by tumor‐derived exosomes and to be associated with immune suppression [52]. These two cell subtypes are both related to the germinal center and represent two stages in a continuous differentiation process. Previous reports have suggested that germinal center B cells can secrete IL‐10 to regulate the quantity and function of Tfr and Tfh cells, which may be a potential mechanism for Breg's inhibition of anti‐infective pathways in PJI [53, 54]. Our findings suggest that CXCR4^+^ PMN‐MDSCs potentially interact with AHR^+^ Bregs via the SEMA4D‐CD72 axis, while the PTPRC‐CD22 pair may mediate their interaction with TIM1^+^ Bregs. Notably, previous studies have established that the engagement of CD22 with the B cell receptor can trigger inhibitory signaling, thereby dampening B cell activation [55]. From a broader physiological perspective, the activation of the Breg/IL‐10 axis in infection represents a ‘double‐edged sword.’ While this regulatory mechanism is essential for preventing hyper‐inflammation and collateral tissue damage during the acute phase of infection, its persistent induction—driven here by CXCR4^+^ PMN‐MDSCs—may become maladaptive in the context of chronic PJI. This shift likely fosters a localized immunosuppressive niche that facilitates ‘immune paralysis,’ ultimately hindering effective pathogen clearance. Although our antibody‐mediated blockade assays provided preliminary evidence for the functional roles of these LR pairs, further in vivo validation is warranted. Elucidating these interactions in more complex physiological settings will be a primary focus of our future investigations. Apart from Bregs and CXCR4^+^ PMN‐MDSCs, the potential interactions between other immunosuppressive cells—such as Tregs, “trojan horse” macrophages, and any immune cells exhibiting immunosuppressive phenotypes—and CXCR4^+^ PMN‐MDSCs or Bregs, as well as their roles in shaping the immunosuppressive network in PJI, remain unresolved issues that warrant further investigation in the future.

Reducing CXCR4^+^ PMN‐MDSCs may be an effective treatment for PJI. Thus, we initially employed AMD3100, a well‐established CXCR4 inhibitor, for treatment, which is known for its ability to hinder the progression and metastasis of diverse tumors [56, 57, 58]. Although AMD3100 has shown some efficacy in treating PJI, its inhibition of CXCR4 is broadly. Beyond CXCR4^+^ PMN‐MDSCs, CXCR4 is also present on certain B cells, T cells, dendritic cells, and others, playing a role in their chemotaxis, migration, and the bone marrow homing of hematopoietic stem cells [59, 60, 61, 62]. It remains uncertain whether inhibiting these processes will alter anti‐infection outcomes, or introduce additional side effects. One study on gastric cancer indicated that while AMD3100 blocked PMN‐MDSC aggregation via the CXCR4/CXCL12 axis, it expedited the release of immature neutrophils from the bone marrow [63]. Hence, we posit that although CXCR4 can be a marker gene for this subgroup, it may not be the most suitable drug target.

Leveraging the global expression profile of CXCR4^+^ PMN‐MDSCs, we utilized oncoPredict and Scissor algorithms to identify alendronate as a potential therapy for CXCR4^+^ PMN‐MDSCs. This screening approach, not reliant on single molecules, effectively mitigates potential risks associated with CXCR4 inhibitors. Alendronate, primarily recognized for osteoporosis treatment, currently lacks robust evidence supporting its bactericidal and anti‐infective properties. Our findings revealed a significant reduction in residual bacterial counts following alendronate monotherapy compared to the infected group. Ignoring alendronate's direct antibacterial effects, we speculate that it likely restores the body's anti‐infective capacity through immune modulation. In mice with skin infections or PJI, alendronate monotherapy notably reduced CXCR4^+^ PMN‐MDSC and Breg levels, enhanced CD8^+^ T cell bactericidal activity, and increased cDC numbers. Notably, although alendronate did not emerge as the primary hit from our initial screening, we recognized the inherent constraints of existing databases. Consequently, we conducted a comprehensive comparative evaluation of pharmacologically related analogs, prioritizing those with optimized efficacy and safety profiles. This refinement process ultimately identified alendronate as our lead candidate, a strategy aimed at selecting compounds with the highest potential for clinical translation and therapeutic utility. Mechanistically, our study identified STAT3 as both a pharmacological target of alendronate and a core TF governing CXCR4^+^ PMN‐MDSCs function. We have functionally validated the role of the CXCR4‐STAT3 axis in driving the interaction between CXCR4^+^ PMN‐MDSCs and Bregs in vitro, accompanied by the upregulation of the suppressive marker ARG1 in the effector PMN‐MDSCs. Notably, through a series of exclusion assays, we demonstrated that alendronate does not exert a direct effect on Bregs. Interestingly, while STAT3 is also a known critical TF for B‐cell function, alendronate appears to lack significant impact on this population. Such cell‐type‐specific effects might be attributed to differences in drug uptake efficiency, endocytic processing, or distinct STAT3‐centered regulatory networks between the two cell types; however, a detailed mechanistic dissection of these differences remains beyond the scope of the present study. In summary, these findings may represent the first reported study on the use of alendronate as an immunomodulatory agent in PJI. Although a more precise mechanism of action remains unanalyzed, it undoubtedly presents a novel strategy for PJI immune regulation. Indeed, alendronate's immunomodulatory effects have been observed in tumor research, reversing T cell function and reducing immunosuppressive factor secretion [64]. The immunomodulatory potential of alendronate warrants further exploration, necessitating more in‐depth mechanistic studies. Additionally, compared to vancomycin monotherapy, the combination of alendronate and vancomycin nearly eradicated all bacteria, suggesting that alendronate may enhance vancomycin's bactericidal effects, potentially representing another anti‐infective mechanism. Currently, alendronate's use in infections primarily focuses on bone‐targeted applications or as a bone resorption inhibitor [65, 66]. Importantly, given the previous literature linking MDSCs to osteoclastogenesis under chronic inflammation, this immunomodulatory capability may extend to the regulation of cell lineage commitment [67, 68]. While this transdifferentiation has primarily been documented in other inflammatory bone loss diseases, it is highly conceivable that under pathological conditions like PJI, MDSCs might similarly possess the potential to differentiate into bone‐resorbing, osteoclast‐like cells, thereby aggravating peri‐prosthetic bone destruction. Consequently, alendronate might exert a dual therapeutic benefit: not only reversing the CXCR4^+^ PMN‐MDSC–mediated immune paralysis but also intercepting their potential differentiation trajectory into osteoclasts. Although the precise occurrence and impact of this process in PJI warrant further experimental validation, this potential cross‐talk introduces a compelling multi‐faceted dimension to its therapeutic utility. It may be time to reevaluate its immunomodulatory effects.

Although this study puts forward an immune regulation strategy centered on abolishing the immune suppression network in PJI, numerous issues still require further exploration in the future. These include how CXCR4^+^ PMN‐MDSCs regulates Bregs through signal crosstalk and whether Bregs can regulate PMN‐MDSCs, too. Additionally, understanding how these cells collaborate with other immunosuppressive cells to construct immune suppression networks is crucial for a more comprehensive grasp of PJI's complex immune microenvironment. Furthermore, while our murine PJI model effectively mimics the localized immune paralysis triggered by bacterial challenge, its inherent limitations—particularly the fundamental interspecies immunological variations and the challenge of fully replicating the chronic disease course seen in clinical settings—must be acknowledged. These constraints necessitate a cautious interpretation of the findings when translating experimental data into clinical therapeutic strategies. Moreover, elucidating how alendronate modulates signal transduction and exerts its immune regulatory effects remains a key challenge, with its specific therapeutic efficacy necessitating evaluation through more rigorous clinical trials. In essence, these represent our future research directions, aiming to offer greater support in the diagnosis and treatment of PJI.

## 4 Conclusions

In our research, CXCR4^+^ PMN‐MDSCs emerged as a novel immunosuppressive cell subtype in the context of PJI, played a role in regulating Breg and demonstrated notable immunosuppressive effects. Its widespread presence in bacterial infections suggests it as a potential therapeutic target for such infections. Alendronate, selected as a targeted drug for CXCR4^+^ PMN‐MDSCs, was used in combination with vancomycin to enhance vancomycin's bactericidal ability, while interfering with the immunosuppressive network and restoring anti‐infective immunity. These findings have been confirmed in mouse models of skin infection and PJI. In conclusion, we have discovered a novel immunosuppressive cell type with high CXCR4 expression and proposed an alendronate‐based immune regulatory strategy, which may be a new prospect in the treatment of bacterial infections.

## 5 Experimental Section

### 5.1 Bacterial Strain and Cell Culture

The MRSA (ATCC 43300) strain was purchase from the American Type Culture Collection and S. aureus strain Xen29 (IVISbrite 119240) was acquired from the PerkinElmer. Following revival from a 37°C water bath, the bacteria were incubated into trypticase soy broth medium and subjected to an overnight incubation at 37°C with shaking at 220 r/min. Prior to each experiment, fresh trypticase soy broth was prepared, and the bacteria were cultured for 4–6 h to attain the logarithmic growth phase.

MC3T3‐E1 (RRID: CVCL_0409) cell line was obtained from Cell Bank of Shanghai Institute of Biotechnology, Chinese Academy of Sciences (Shanghai, China). All cell lines were confirmed to be free of contamination and were maintained in good condition. MC3T3‐E1 cells were cultured in α‐MEM medium supplemented with 10% FBS, 100 U/mL penicillin, and 100 U/mL streptomycin (PS).

### 5.2 Animals and Model Construction

Male C57BL/6J mice of specific pathogen‐free grade, aged between 6 to 8 weeks, were procured from JieSiJie Biotechnology Co., Ltd (Shanghai, China). Prior to establishing the model, permit the mice to acclimate to their surroundings for a period of 3 to 5 days, ensuring they have unrestricted access to both water and food. All animal experimental procedures carried out in vivo were authorized by the Animal Ethics Committee of Shanghai YiShang Biotechnology Co., Ltd (Shanghai, China) and performed following the guidelines of the National Institutes of Health guide for the care and use of Laboratory Animals.

For murine skin infection model, the mice were rendered unconscious through an intraperitoneal administration of 1% pentobarbital sodium at a dosage of 10 mL/kg, after which their backs were shaved to reveal the underlying skin. To enable continuous monitoring of bacterial load, mice were administered subcutaneous injections of 200 µl of Xen29 S. aureus solution (OD600 = 0.2, 10^7^ CFU/mL) on both sides of their bodies. 2 h post‐infection, treatments were initiated according to the respective experimental groups: the vehicle group (PBS); the vancomycin group (80 mg/kg, i.p.); the alendronate group (0.25 mg/kg s.c); and the combined group (80 mg/kg vancomycin i.p. + 0.25 mg/kg alendronate s.c). Subsequent treatments were carried out every 24 h.

For the PJI model, sterile titanium rods with dimensions of 0.6 mm × 6–8 mm were prepared prior to surgery. Following fur removal and skin disinfection around the right knee joint of the mouse, a 1 cm incision is made along the medial patellar side to expose the knee joint. The hole is then carefully enlarged from the distal end of the femur along its longitudinal axis toward the proximal end, and a sterile titanium rod is inserted. Subsequently, 2 µl of MRSA solution (OD600 = 0.2, 10^7^ CFU/mL) is added to the titanium rod's terminus, followed by layer‐by‐layer suturing. After 7 days of infection, treatment is administered according to the experimental grouping.

### 5.3 Single‐Cell Suspension Preparation and scRNA Sequencing

For comprehensive cell analysis, soft tissues surrounding the right knee joint were obtained from both PJI model mice and their control counterparts. These tissues were thoroughly washed three times with sterile PBS and subsequently minced into small tissue fragments. Subsequently, the tissue pieces were immersed in a pre‐prepared digestion mixture containing Collagen I (1.25 mg/mL), dispersing enzyme (1 mg/mL), and DNase I (0.05 mg/mL). The samples were then incubated on a shaking table at 37°C for 30 min. After digestion, a 70 µm filter was utilized to eliminate any remaining undigested tissue components. The cell suspension was centrifuged at 600 g for 5 min to collect cells, which were then rinsed twice with sterile PBS. Finally, cell viability was assessed to confirm that it surpassed the 90% threshold.

For sorted MDSCs, following tissue digestion, red blood cells were lysed. Next, the Zombie Aqua Stain with Fixable Viability Kit (423102, Biolegend) was applied for 15 min, followed eliminate free reagents. After blocking non‐specific Fc receptor binding with an anti‐mouse CD16/32 antibody (156603, Biolegend), the cells were stained for MDSCs using PerCP/Cy5.5 anti‐mouse CD45 (103132, Biolegend), APC/Fire750 anti‐mouse CD11b (101262, Biolegend), and BV605 anti‐mouse Gr1 (108439, Biolegend) antibodies. Finally, cell sorting was performed using the BD FCAria III system.

In strict accordance with the operational guidelines outlined in the 10 × Genomics Chromium Next GEM Single Cell 3ʹ Reagent Kits v3.1 (PN‐1000268), the single‐cell suspension was carefully loaded onto the instrument, and subsequent library construction was carried out. The constructed library was subjected to high‐throughput sequencing using the Illumina Nova 6000 PE150 platform.

### 5.4 scRNA Sequencing Data Processing

Raw sequencing data underwent analysis via CellRanger software for alignment to the Mus musculus reference genome (GRCm38/mm10), followed by cell filtering and gene‐level quantification. The resulting filtered expression matrix was analyzed with the Seurat package (version 4.2.2).

Quality control was performed by retaining cells with: (1) unique feature counts between 300 and 5000, and (2) mitochondrial gene content <10% of total UMI counts. Data normalization was conducted using SCTransform with default parameters. Doublet identification and removal were performed using DoubletFinder (version 2.0.3) with an expected doublet rate based on the cell number in each sample. Batch effects were corrected using the Harmony package (version 1.1.0). For dimensionality reduction, the first 15 principal components were selected based on elbow plot analysis and used for both Uniform Manifold Approximation and Projection (UMAP) visualization and graph‐based clustering at a resolution of 0.5. Differential gene expression analysis was performed using the FindAllMarkers function to identify cluster‐specific marker genes. Cell types were annotated based on the top differentially expressed genes and canonical markers from literature.

### 5.5 Gene Sets Scoring

To evaluate the cell population within the PMN subgroup that functions more similarly to MDSCs, the AUCell package was employed to score the PMN‐MDSC gene set. To ensure more rigorous conclusions, three PMN‐MDSC gene sets, all sourced from previous literature, were utilized [13, 69]. The gene set related to PMN functions was derived from research on 12 characteristic gene sets, encompassing chemotaxis, NADPH oxidase, phagocytosis function, etc [70]. The scoring results calculated from the AUCell package were visualized using a box plot.

### 5.6 Cell Trajectory Analysis

Monocle3 represents an efficient approach for pseudotime analysis [71]. The new_cell_data_set function is employed to construct CDS objects. Following data preprocessing with the preprocess_cds function, the learn_graph functions are applied to cluster the cells and infer their developmental trajectory, respectively. Ultimately, the differentiation trajectory is visualized based on the original UMAP graph of PMN subgroups. The color gradient transitioning from purple to yellow signifies the developmental trajectory of cells from an immature to a mature state.

To validate the differentiation outcomes, Slingshot analysis was utilized to deduce the differentiation trajectory of PMN subgroups [72]. The slingshot function is responsible for executing the primary trajectory analysis.

### 5.7 Differential Expression and Enrichment Analysis

In order to identify the various function between cell types, the “FindMarkers” function was applied. The differential expression results of all genes were utilized as input data for GO enrichment analysis, which was conducted based on Gene Set Enrichment Analysis (GSEA). A p‐value less than 0.05 was regarded as statistically significant.

### 5.8 Cell‐Cell Interaction Analysis

The Cellchat R package is a classic tool for cell communication analysis [73]. It first calculates the count and weight of cell communication signals, and then compares the differences in incoming and outgoing signals between uninfected and infected groups across various cell types. Subsequently, it evaluates the cell types that exhibit the closest communication with the subtype cells of interest and visualizes these relationships using the CCPlotR package [74]. The ligand‐receptor (LRs) involved in cell communication are sourced from a built‐in database, and a bubble plot is used to display the communication probability scores of the LRs participating in cell interactions.

### 5.9 SCENIC Analysis

To identify the core transcription factors (TFs) and their underlying gene regulatory networks driving PMN subpopulations, we performed SCENIC analysis. Briefly, co‐expression modules between TFs and candidate target genes were initially inferred using the GRNBoost2 algorithm. Subsequently, these modules were refined via cis‐regulatory motif enrichment analysis using RcisTarget to retain only direct targets, thereby constructing highly confident regulons. Finally, the regulon activity score for each individual cell was quantified using the AUCell algorithm. Core TFs defining specific PMN subtypes were identified by comparing the differential regulon activities across distinct clusters.

### 5.10 Public Datasets Collection and Process

To illustrate the pervasive presence of CXCR4^+^ PMN‐MDSC in bacterial infections, we collected three distinct types of single‐cell infection datasets from the Gene Expression Omnibus (GEO) and Zenodo databases. These datasets include GSE237646 (containing 3 samples of Pseudomonas aeruginosa (P. aeruginosa) infection), GSE137539 (containing 6 samples of Escherichia coli (E. coli) infection), and the sepsis dataset published by Knight et al. (containing 39 sepsis samples). The data processing procedure is in line with that applied to the PJI sequencing data described earlier. PMNs were identified through screening using well‐established biomarkers, namely S100A8, S100A9, CSF3R, LCN2, IL1R2. Subsequently, these cells underwent secondary dimensionality reduction clustering. A density plot was drawn to exhibit biomarkers’ expression levels.

Additionally, we gathered bulk RNA sequencing data to underscore the significance of MDSCs in bacterial infections. All the bacterial infection‐related bulk RNA datasets were sourced from the GEO database. These include GSE255786 (comprising 40 aseptic failure (AF) patients and 53 PJI patients), GSE13015 (comprising 77 infected tissues and 8 control tissues), GSE25504 (comprising 28 infected tissues and 35 control tissues), GSE33341 (comprising 51 infected tissues and 43 control tissues), GSE4607 (comprising 52 infected tissues and 15 control tissues), and GSE69528 (comprising 83 infected tissues and 55 control tissues). When assessing the proportion of CXCR4^+^ PMN‐MDSC in infections caused by different bacterial species, to enlarge the sample size of each group, we merged the datasets containing bacterial species information and removed their batch effects. Specifically, we employed the ComBat function from the sva package to eliminate batch effects. We conducted principal component analysis (PCA) both before and after batch effect removal to evaluate the efficacy of this process.

Given that sepsis is predominantly caused by bacterial infections, we also collected a partial sepsis bulk RNA dataset to assess the relationship between CXCR4^+^ PMN‐MDSC and prognosis. This dataset comprises GSE137340 (including 48 survivors and 3 non survivors), GSE185263 (including 293 survivors and 52 non survivors), GSE26378 (including 91 survivors and 12 non survivors), GSE26440 (including 99 survivors and 17 non survivors), GSE54514 (including 96 survivors and 31 non survivors), GSE65682 (including 365 survivors and 114 non survivors), and GSE95233 (including 68 survivors and 34 non survivors). All the data were normalized using the limma package.

### 5.11 Deconvolution of Bulk RNA Data Using scRNA Sequencing Data

The deconvolution algorithm is capable of inferring high‐dimensional compositional information from low‐dimensional observations by utilizing a reference dataset. To calculate the fraction of PMN subgroups in public datasets, the IOBR package carried out a deconvolution process [75]. Specifically, the generateRef_deurat function extracted the feature gene expression matrix of each cell subpopulation from scRNA sequencing data to construct a reference gene set, and the decorvo_tme function performed deconvolution analysis based on the Support Vector Regression algorithm.

### 5.12 Drug Screening Targeting CXCR4^+^ PMN‐MDSC

Considering that virtual screening based on molecular markers may lack precise cell‐specific targeting, we adopted an alternative, potentially more targeted approach for drug screening. First, the AverageExpression function constructs the feature expression matrix for each PMN subgroup in each sample. The babelgene package converts mouse gene names to Homo sapiens gene names, and the calcPhenotype function in the oncoPredict package conducted drug sensitivity analysis [76]. We extract the IC50 results of CXCR4^+^ PMN‐MDSCs, and perform differential IC50 analysis between uninfected and infected groups, and select the drug with the smallest logFC value as the final candidate drug under the significance condition of p value < 0.05.

The Scissor algorithm tests the cell‐specific targeting of candidate drugs. Specifically, using bulk sequencing data of drug‐treated cell lines from the GDSC2 database as input, samples are divided into drug‐sensitive and insensitive groups based on the median IC50 value of the candidate drug. The Scissor function performed integration analysis of cell subpopulations, where Scissor^+^ represented cells sensitive to the candidate drug.

### 5.13 Target Fishing and Molecular Docking

The structure of alendronate was retrieved from the PubChem database and imported into ChemBio3D 14.0 software for spatial conformation adjustment and energy optimization. It was then imported into the COMET human protein database for reverse docking to identify potential targets. Proteins with high confidence were selected and ranked accordingly.

Then, we utilized the RCSB PDB database to screen for STAT3 protein targets with high‐resolution crystal structures, which will serve as molecular docking receptor. After that, PyMOL software was used to perform operations such as dehydration and phosphate removal on the protein. Finally, AutoDock Vina 1.5.6 software was employed for molecular docking to obtain the optimal conformation.

### 5.14 Molecular Dynamic Simulation

Gromacs 2022 software is employed to investigate the dynamic interactions within the alendronate‐STAT3 complex. Specifically, receptor protein uses Amber14SB force field, while ligand uses GAFF2 force field for parameterization. Ions are added with gmx genion command for neutrality. All critical bond constraints are imposed via the SHAKE algorithm. The energy minimization procedure encompasses 3000 iterations employing the steepest descent approach, followed by 2000 iterations utilizing the conjugate gradient technique to achieve optimal system refinement. Radius of gyration (Rg), solvent accessible surface area (SASA), and root mean square fluctuation (RMSF) are calculated with g‐Rg, g‐sasa, and g_rmsf.

### 5.15 Protein Interaction Pull‐Down Assay

In the pull‐down experiment, adhere to the guidelines provided by the PierceTM Biotinylated Protein Interaction Pull‐Down Kit (21115, Thermo Fisher). Specifically, incubate biotinylated alendronate with streptavidin agarose beads at 4°C for 30 min, utilizing biotin as the control. Following this, introduce the sorted CXCR4^+^ PMN‐MDSC lysate overexpressing STAT3 and gently agitate the mixture with the beads for 24 h. The subsequent day, perform three washing process, add the elution buffer, and then wash in a 95°C water bath. Separate the protein samples using 4%–12% sodium dodecyl sulfate polyacrylamide gel electrophoresis (SDS‐PAGE), with the total lysates serving as input controls.

### 5.16 Hemolysis Assay

The method for detecting the hemolytic activity of alendronate acid was adapted from existing literature [77]. In brief, fresh mouse venous blood, which had been heparinized, was centrifuged at 2000 g for 10 min. The red blood cells were then washed five times with PBS and adjusted to the appropriate concentration. Subsequently, an equal volume (100 µl) of the red blood cell suspension was combined with a twofold serial dilution of alendronate prepared in PBS, and this mixture was added to a 96‐well plate. For control purposes, 0.2% DMSO was used as the negative control, while 1% Triton X‐100 served as the positive control. Following an incubation at 37°C, the plate was centrifuged at 1000 g for 10 min. Then, 100 µL of the supernatant was transferred to a fresh plate, and its absorbance was measured at 540 nm.

### 5.17 Magnetic Bead‐Based Isolation of CXCR4^+^ PMN‐MDSCs

To isolate CXCR4^+^ PMN‐MDSCs, periarticular soft tissues of the PJI mouse knee joints were digested according to the aforementioned tissue digestion protocol. The resulting digest was filtered through a 40‐µm cell strainer to obtain a single‐cell suspension. PMN‐MDSCs were initially isolated using the Myeloid‐Derived Suppressor Cell Isolation Kit (130‐094‐538, Miltenyi Biotec) following the manufacturer's instructions. Subsequently, the sorted PMN‐MDSCs were labeled with a PE‐conjugated anti‐CXCR4 antibody. Finally, the CXCR4^+^ PMN‐MDSC subpopulation was further enriched using the EasySep Mouse PE Positive Selection Kit II (17656, StemCell Technologies).

### 5.18 Cell Co‐Culture Assays

To evaluate the suppressive effect of CXCR4^+^ PMN‐MDSCs on T cells, mouse splenocytes were harvested, and T cells were isolated using the EasySep Mouse T Cell Isolation Kit (Cat#19851, StemCell) according to the manufacturer's instructions. The isolated T cells were activated in 96‐well plates pre‐coated with anti‐CD3 and anti‐CD28 antibodies. For the proliferation assay, T cells were labeled with 10 µM eFluor670 (Cat#65‐0840‐85, Thermo Fisher Scientific) for 10 min prior to co‐culture. CXCR4^+^ PMN‐MDSCs and T cells were then co‐cultured at a 1:1 ratio for 4 days.

To investigate the induction of Breg cells by CXCR4^+^ PMN‐MDSCs, B cells were isolated from mouse splenocytes using the EasySep Mouse B Cell Isolation Kit (Cat#19854, StemCell). The B cells were pre‐stimulated with 5 µg/mL anti‐IgM and 2 µg/mL anti‐CD40 for 6 h. Subsequently, CXCR4^+^ PMN‐MDSCs and B cells were co‐cultured at a 1:1 ratio for 24 h.

For the detection of Granzyme B (GZMB) and IFN‐γand (IFNG) secretion and Breg cell frequencies, the co‐cultured cells were stimulated with Cell Activation Cocktail for 4–6 h prior to flow cytometry.

### 5.19 Adoptive Transfer

To evaluate the specific role of CXCR4^+^ PMN‐MDSCs in modulating Breg cells, endogenous PMN‐MDSCs were first depleted in vivo according to a previously established protocol [78]. Briefly, one day prior to the induction of the PJI model, mice received an i.p. of an anti‐Ly6g neutralizing antibody (Clone 1A8; BioXCell) at a loading dose of 400 µg. Subsequently, maintenance doses of 100 µg were administered i.p. three times per week until the PJI model was successfully established. Following depletion and model establishment, mice were randomly assigned to receive adoptive transfers as previous study [79]. Purified CXCR4^−^ PMNs or CXCR4^+^ PMN‐MDSCs (2 × 10^5^ cells/mouse) were resuspended in 150 µL of sterile PBS and injected intravenously into the recipient mice. At the designated experimental endpoint, all recipient mice were euthanized, and the relevant tissues were harvested for further analysis.

### 5.20 Real‐Time Quantitative Polymerase Chain Reaction (RT‐qPCR)

Total RNA from cells was extracted using the RNAsimple Total RNA Kit (RC102‐01, Vazyme) according to the manufacturer's instructions. RNA purity was assessed using a NanoDrop spectrophotometer (Thermo Fisher Scientific, USA). Genomic DNA was removed prior to reverse transcription. cDNA synthesis was followed by quantitative real‐time PCR performed on the QuantStudio5 platform using SYBR qPCR Master Mix (Q713‐02, Vazyme). GAPDH served as the internal control. Each gene was analyzed in triplicate, and relative expression levels were calculated using the 2^–ΔΔCt method. Primer sequences are provided in Table S1.

### 5.21 Western Blotting

Total protein was extracted using RIPA lysis buffer supplemented with protease and phosphatase inhibitors. After washing with ice‐cold PBS, cells were lysed on ice, and the supernatant was collected via centrifugation at 12000 rpm for 10 min. Protein concentrations were quantified using a BCA protein assay kit. Subsequently, samples were normalized, diluted with 5× loading buffer, and denatured at 100°C for 10 min. Equal amounts of protein were separated by SDS‐PAGE and then wet‐transferred onto ethanol‐activated polyvinylidene fluoride membranes. After blocking with rapid blocking buffer, the membranes were incubated with primary antibodies overnight at 4°C. After washing, the membranes were incubated with secondary antibodies at room temperature. Finally, the protein bands were visualized using enhanced chemiluminescence reagents

### 5.22 Flow Cytometry and Fluorescence Activated Cell Sorter

Tissue dissociation and the preparation of a single‐cell suspension were carried out as described previously. A 70 µm filter was used to filter the suspension, thereby removing any undigested tissue fragments. Following red blood cell lysis, the cells were pelleted by centrifugation at 600 × g for 5 min. The viability of the cells was then assessed using the Zombie Aqua Fixable Viability Kit (BioLegend, 423101) to distinguish between live and dead cells. Subsequently, Fc receptors were blocked using an anti‐mouse CD16/32 antibody (BioLegend, 156603). Cells were then stained with cell surface markers.

To evaluate intracellular factors, including GZMB, freshly isolated tissue single‐cell suspensions or co‐cultured T cells were maintained in complete RPMI‐1640 medium (10% FBS, 1% P/S, and 50 µM β‐mercaptoethanol) at 37°C in a humidified incubator with 5% CO_2_. To induce and retain intracellular proteins, tissue single‐cell suspensions were stimulated with Cell Activation Cocktail containing Brefeldin A (423303, BioLegend) for 6 h; for the co‐culture group, the same cocktail was added during the final 6 h of co‐culture. Post‐stimulation, cells were harvested and subjected to cell surface marker staining as described above. For subsequent intracellular staining, cells were fixed and permeabilized using the Fixation/Permeabilization Buffer (00‐5523‐00, eBioScience) for 30 min. Cells were then incubated with fluorochrome‐conjugated antibodies in 1× permeabilization buffer for 30 min at 4°C. All antibodies used in flow cytometry were described in Table S2.

For functional assays, cell sorting was performed using a BD FACSAria III equipped with a 100 µm nozzle at low pressure to maintain cell viability. Unlike analytical flow cytometry, sorted cells were collected into pre‐chilled tubes containing complete culture medium (DMEM or RPMI‐1640 with 20% FBS) to minimize post‐sort stress and ensure downstream experimental success.

### 5.23 IL‐10 Measurement

Quantification of IL‐10 levels within culture supernatants was performed using the Mouse IL‐10 Valukine ELISA Kit (VAL605, Bio‐Techne), strictly adhering to the manufacturer's guidelines. The absorbance was measured at a wavelength of 450 nm using a microplate reader, and the IL‐10 concentrations were subsequently calculated based on the standard curve.

### 5.24 Histological Assessment

Skin tissues and the soft tissues surrounding the knee joint were harvested from various groups. These tissues were promptly fixed in a 4% paraformaldehyde solution for a duration of 48 h to preserve their cellular and tissue architecture. Following fixation, the tissues underwent a sequential dehydration process using ethanol solutions with a gradient of ethanol. Tissues were embedded in paraffin wax to ensures their structural integrity is maintained for subsequent histological analysis.

Similarly, the femurs were collected from PJI mice. After fixation with 4% paraformaldehyde, the femur was decalcified using 10% EDTA solution (Servicebio, Wuhan, China) for 2 weeks, dehydrated using an alcohol gradient, and then embedded in paraffin.

Hematoxylin and eosin (HE) staining was employed to visualize the overall tissue morphology and identify areas of inflammation. Immunohistochemical staining was utilized to quantitatively assess the protein expression levels of key inflammatory markers, specifically interleukin‐1 beta (IL1B) and tumor necrosis factor‐alpha (TNF‐α). Additionally, tartrate‐resistant acid phosphatase (TRAP) staining was performed to evaluate the extent of bone resorption and osteoclast activity by carefully observing the morphology and counting the number of osteoclasts present in the tissue sections.

### 5.25 Immunofluorescent Staining

Paraffin sections (approximately 2 µm) were prepared from the embedded tissue samples. Following antigen retrieval to unmask hidden epitopes, endogenous peroxidase activity was quenched by treating the sections with a 3% hydrogen peroxide solution. 5% goat serum was used to block the non‐specific binding at room temperature for 30 min. Next, the slides were incubated overnight at 4°C with primary antibodies, including anti‐Gr1 (108401, Biolegend), anti‐Cxcr4 (60042‐1‐Ig, proteintech), anti‐Ly6g, anti‐Cd19, anti‐Ahr (28727‐1‐AP, Proteintech), anti‐Tim1 (RMT1‐10, Invitrogen), anti‐Col1a1, and anti‐S. aureus (ab20920, abcam). After rinsing away the unbound primary antibody, the slides were treated with a fluorescently conjugated secondary antibody for 1 h at room temperature. Finally, each slide was mounted using a DAPI‐containing, wear‐resistant mounting medium. The staining results were then captured using confocal microscopy, which offers High Power Field (HPF) for statistics.

### 5.26 Bioluminescence Detection

To monitor the bacterial load at various infection time points in real‐time, a live imaging system (PerkinElmer, USA) was employed to measure the bioluminescence intensity of Xen29. Prior to the experiment, the back hair of the mice was shaved cleanly. During the imaging process, anesthesia was maintained using isoflurane to ensure the mice remained still. The imaging settings were adjusted according to the system's default parameters for bioluminescence detection. For data collection, the average signal value from both sides of each mouse was recorded as a single data point, and statistical analysis was performed using Avg Radiance

### 5.27 Radiographic Evaluation

X‐rays can observe the position of implants in the femur. Radiographic assessments were carried out in line with established standards [80]. The evaluation encompassed five key dimensions: (i) elevation of the periosteum, (ii) broadening of the bone shaft, (iii) distortion of bone architecture, (iv) alterations in soft tissue, and (v) formation of new bone. Each of these dimensions was graded on a scale from 0 to 4, where a score of 4 represented the most severe manifestation.

### 5.28 Micro‐CT Assessment

To thoroughly evaluate bone resorption and regeneration processes, we utilized a high‐resolution microCT scanner (SkyScan 1072; Kontich, Belgium). The femur underwent scanning with this device, operating at a voltage setting of 70 kV and a current of 200 µA. The assessment of osteolysis and bone regeneration primarily relied on five key parameters: (i) trabecular bone mineral density (Tb.BMD), (ii) the ratio of bone volume to tissue volume (BV/TV), (iii) trabecular thickness (Tb.Th), (iv) the degree of separation between bone trabeculae (Tb.Sp), and (v) the structural model index (SMI).

### 5.29 Statistical Analysis

Data processing, statistical analysis, and visualization are all conducted utilizing the R software (version 4.2.2). The data are presented as the mean ± standard deviation. Initially, normality and variance homogeneity tests are done; t‐test for normal, homogeneous groups; Wilcoxon test for skewed, heterogeneous groups.

For immunofluorescence co‐localization analysis, the JACoP plugin within ImageJ software is used. Prior to analysis, background noise is subtracted from the images, and a fluorescence signal threshold is established. Co‐localization is evaluated based on Pearson's Correlation Coefficient, with a value approaching 1 indicating stronger co‐localization.

The immunohistochemical score is calculated by multiplying the positive intensity by the positive cell ratio. The positive intensity is graded on a scale of 0–3, representing no positive, weak positive, moderate positive, and strong positive staining, respectively. The positive cell ratio is categorized into five levels (0–4), corresponding to 0%–5%, 6%–25%, 26%–50%, 51%–75%, and >75% positive cells, respectively.

Inter‐group differences are considered statistically significant at *p* < 0.05, with significance levels denoted as * *p* < 0.05, ** *p* < 0.01, and *** *p* < 0.001.

## Author Contributions

J. W., S. Z., and Y. L. performed experiments, analyzed data and visualization. J. W., S. Z., J. J., Z. F., and Q. H. contributed to the animal experiments. Z. Z., Y. W. contributed to the literature search. J. W., S. Z., and Y. L. wrote the manuscript. B. Y., F. S., and X. Q. contributed to the study design and revised the manuscript.

## Ethics Statement

All animal experimental procedures carried out in vivo were authorized by the Animal Ethics Committee of Shanghai YiShang Biotechnology Co., Ltd (IACUC issue No. IACUC‐2025‐I‐017) and performed following the guidelines of the National Institutes of Health guide for the care and use of Laboratory Animals.

## Conflicts of Interest

The authors declare no conflict of interest.

## Supporting information




**Supporting File**: advs76149‐sup‐0001‐SuppMat.docx.

## Data Availability

The public dataset employed in this study originates from the GEO database and zenodo, with the specific accession numbers as follows: For the scRNA dataset, it comprises the sepsis‐related dataset generated by Knight et al., GSE237646, and GSE137539. The Bulk RNA dataset encompasses bacterial infection‐related datasets from mice, namely GSE13015, GSE25504, GSE33341, GSE4607, and GSE69528, along with PJI or sepsis‐related datasets from homo sapiens, including GSE255786, GSE137340, GSE185263, GSE26378, GSE26440, GSE54514, GSE65682, and GSE95233. The scRNA sequencing data newly generate in this study would be available upon reasonable request.
