# Supplementary material for: Targeting a Myeloid–Regulatory B Cell Network Reverses Immune Paralysis in Periprosthetic Joint Infections
Source: Adv Sci (Weinh). 2026 Jun 22;13(51):e76149. doi: 10.1002/advs.76149 (PMC13336358; doi:10.1002/advs.76149)
Supplement: Supplementary file 1 [file ADVS-13-e76149-s001.docx]

Supporting Information

Targeting a myeloid–regulatory B cell network reverses immune paralysis in periprosthetic joint infections

Jintao Wu^a#^, Shutao Zhang^a#*^, Yumin Lin^c#^, Juyang Jiao^d^, Zhiwei Fu^a^, Qimin Hong^a^, Ziyi Zhao^a^, Xinhua Qu^a*^, Fei Su^b*^, Bing Yue^a*^

**Supplementary Information Legends**

**Figure S1. PMN-MDSCs Play a Key Role in Immunosuppression throughout the Different Stages of PJI, related to Figure 1**

**Figure S2. PMN-MDSCs in PJI Exhibit Characteristic High Expression of CXCR4, related to Figure 2**

**Figure S3. Assessment of T Cell Immunosuppression and Proliferation, related to Figure 2**

**Figure S4. CXCR4⁺ PMN-MDSCs Are Widely Detected During Bacterial Infections**

**Figure S5. CXCR4⁺ PMN-MDSCs Are Correlated with Prognosis in Sepsis**

**Figure S6. CellChat Analysis of the Major Cell Types in the Knee Joint Soft Tissue of Mice**

**Figure S7. Identification and Functional Characterization of Distinct Breg Subsets in Uninfected and Infected Mice, related to Figure 3**

**Figure S8. CXCR4^+^ PMN-MDSCs Interact with Bregs to Form an Immunosuppressive Network in PJI, related to Figure4**

Figure S9. Expression of CXCR4 in Different Cell Types, related to Figure 5

Figure S10. Alendronate Blocks the Immunosuppressive Network by Regulating CXCR4^+^ PMN-MDSC, related to Figure 6 and Figure 7

Figure S11. Drug Safety of Alendronate

Figure S12. Alendronate Combined with Vancomycin Attenuates Skin Infection by Reversing Immunosuppression, related to Figure 8

Figure S13. Micro-CT results of Different Treatment, related to Figure 9

Figure S14. H&E and Immunohistochemical Staining of the Femurs in PJI Mice, related to Figure 9

Figure S15. FC Analysis Results of Immune Cells in the Knee Joint Soft Tissue of PJI Mice, related to Figure 9

**Table S1.** **Primers of qRT-PCR**

**Table S2.** **Information of Reagents and Primary Antibodies**

**Figure S1. PMN-MDSCs Play a Key Role in Immunosuppression throughout the Different Stages of PJI, related to Figure 1**

(**A-D**) GSVA score of anti-bacterial-related pathway between the uninfected and infected groups in GSE25504, GSE33341, GSE4607, GSE69528, respectively. (**E**) Statistics analysis of M-MDSC proportion (n=5 per group). (**F-G**) PMN-MDSC and M-MDSC fraction between the survivor and non-survivor groups in GSE4607. (Bar plot displays the means ±SD, box plot displays the mean value; ns = not significant, *p < 0.05, **p < 0.01, ***p < 0.001).

**Figure S2. PMN-MDSCs in PJI Exhibit Characteristic High Expression of CXCR4, related to Figure 2**

(**A-B**) UMAP plot and marker gene dot plot for the major cell types identified by scRNA sequencing. (**C**) The stacked bar chart shows the proportion of different cell types in the uninfected and infected groups. (**D**) Marker gene dot plot for PMN subtypes. (**E**) The stacked bar chart shows the proportion of PMN subtypes in the uninfected and infected groups. (**F-G**) Using two additional MDSC gene sets for GSVA scoring validation of PMN-MDSC signature for PMN subgroups. (**H**) Boxplot of activity scores of various functions of PMN, including Cytoplasmic granules, chemotaxis, NADPH oxidase, phagocytosis and metabolic processes.

**Figure S3. Assessment of T Cell Immunosuppression and Proliferation, related to Figure 2**

(**A**) Flow cytometry detection of GZMB and IFNG secretion in activated T cells co cultured with CXCR4^-^ PMN or CXCR4^+^ PMN-MDSC. (**B**) Proliferation detection in activated T cells co cultured with CXCR4^-^ PMN or CXCR4^+^ PMN-MDSC(n=5 per group). (Box plot displays the mean value; ***p < 0.001).

**Figure S4. CXCR4⁺ PMN-MDSCs are Widely Detected During Bacterial Infections**

(**A-C**) UMAP and density plots revealing CXCR4^+^ PMN-MDSC presence across different bacterial infection datasets. (**D-F**) Density plots for representative PMN-MDSC genes in different bacterial infection datasets. (**G-I**) UMAP and density maps, based on scRNA sequencing of flow cytometry-sorted Gr1^+^ cells, show PMN subpopulation distribution and the expression of CXCR4 and SLC2A1. (**J**) Violin plot for the PMN-MDSC signature in PMN subtypes.

**Figure S5. CXCR4⁺ PMN-MDSCs Are Correlated with Prognosis in Sepsis**

(**A-B**) PCA of the combined bulk dataset related to bacterial infections, both before and after batch effect removal. (**C**) Evaluation of CXCR4^+^ PMN-MDSC relative abundance in datasets for various types of bacterial infections. (**D-E**) The relationship between the fraction of CXCR4^+^ PMN-MDSC and both survival and PRISM score in GSE4607. (**F**) The MDSC fraction between the survivor and non-survivor groups in different sepsis datasets. (**G**) The relationship between the fraction of CXCR4^+^ PMN-MDSC and SOFA score in sepsis dataset (GSE185263). (Box plot displays the mean value; *p < 0.05, **p < 0.01, ***p < 0.001).

**Figure S6. CellChat Analysis of the Major Cell Types in the Knee Joint Soft Tissue of Mice**

(**A**) The count and weight of cell communication of CXCR4^+^ PMN-MDSC. (**B**) The incoming and outgoing signaling between CXCR4^+^ PMN-MDSC and other cells. (**C**) Comparison of cell-cell communication counts between the uninfected group and the infected group. (**D**) Comparison of cell-cell communication weights between the uninfected group and the infected group. (**E**) Density plot for the expression of Cxcl12 from the scRNA sequencing data. (**F**) Bubble plot showing ligand-receptor interactions of CXCL signaling received by CXCR4^+^ PMN-MDSC.

**Figure S7. Identification and Functional Characterization of Distinct Breg Subsets in Uninfected and Infected Mice, related to Figure 3**

**(A**) Marker gene dot plot for B cell subtypes. (**B**) Proportion of Breg subtypes in control and SA group. (**C-E**) GOBP enrichment analysis of 3 types of Bregs. (**F**) Estimation of Breg subtype abundance differences in the human PJI cohort GSE255786 using deconvolution algorithms (AF=40, PJI=53). (**G**) Flow sorting gate logic diagram of Breg subtypes. (**H-I**) Flow cytometry analysis of the effects of different Breg subgroups on IFNG secretion in activated T cells (n=5 per groups). (Box plot displays the mean value; *p< 0.05, ***p < 0.001).

**Figure S8. CXCR4^+^ PMN-MDSCs Interact with Bregs to Form an Immunosuppressive Network in PJI, related to Figure4**

(**A-B)** Validation of the depletion effect of anti-Ly6g on PMN-MDSCs by flow cytometry (n=5 per group). (**C**) CXCR4^+^ PMN-MDSC flow sorting logic diagram. (**D**) Flow cytometry displays the effects of depleted PMN-MDSCs and adoptive transfer of CXCR4^-^ PMNs or CXCR4^+^ PMN-MDSCs on TIM^+^ Bregs. (Box plot displays the mean value; ***p < 0.001).

**Figure S9. Expression of CXCR4 in Different Cell Types, related to Figure 5**

(**A**) A feature plot of the expression of CXCR4 in different cell types. (**B**) A violin plot of the expression of CXCR4 in different cell types.

**Figure S10. Alendronate Blocks the Immunosuppressive Network by Regulating CXCR4^+^ PMN-MDSC, related to Figure 6 and Figure 7**

(**A**) Apoptosis percentage of MC3T3-E1 treated with serial dilution of alendronate. (**B-D**) Molecular dynamic simulation results of the Rg, SASA, and RMSF between alendronate and STAT3. (**E**) ELISA detection of the effect of alendronate on B cell IL-10 secretion (n=5 per groups). (**F-G**) WB demonstrates the effect of alendronate on p-STAT3 in Breg (n=3 per groups). (Box plot displays the mean value; ns = not significant, **p< 0.01, ***p < 0.001).

**Figure S11. Drug Safety of Alendronate**

(**A**- **B**) Drug hemolysis assessment of Alendronate, Percentage hemolysis = (OD540nm of felodipine − OD540nm of DMSO) / (OD540nm of TritonX-100 − OD540nm of DMSO) × 100. (**C-F**) Serum levels of AST, ALT, IL-6, and IL-10 in PJI mice following treatment with PBS, vancomycin, alendronate, or their combination. (**G**) H&E staining of heart, kidney, liver, lung, and spleen in mice with PJI following treatment with PBS, vancomycin, alendronate, or both together (scale bar: 100μm). (Box plot displays the mean value; ns = not significant, *p< 0.05).

**Figure S12. Alendronate Combined with Vancomycin Attenuates Skin Infection by Reversing Immunosuppression, related to Figure 8**

(**A**) H&E staining reveals the morphological characteristics of the inflammatory region in skin infections of day 1 and day3 in mice with PJI following treatment with PBS, vancomycin, alendronate, or both together (scale bar: 100μm). (**B**) Immunohistochemical staining of IL-1β in skin infections of day 1 and day3 in the indicated groups (scale bar: 100μm). (**C**) Representative immunofluorescence images of CXCR4^+^ PMN-MDSC in skin infections of day 1 and day3 in the indicated groups (scale bar: 10μm).

**Figure S13. Micro-CT results of Different Treatment, related to Figure 9**

(**A**) Statistical analysis of micro-CT parameters of femurs in PJI mice treated with PBS, vancomycin, alendronate, or both together, including Tb.BMD, Tb.BV/TV, Tb.Th, Tb.Sp, and SMI (n=5 per groups). (B) Statistical analysis of micro-CT parameters of femurs in PJI mice treated with PBS, anti-Ly6g, or a combination of anti-Ly6g and alendronate. (Box plot displays the mean value; ns = not significant, **p< 0.01, ***p < 0.001.)

**Figure S14. H&E and Immunohistochemical Staining of the Femurs in PJI Mice, related to Figure 9**

(**A**) H&E staining reveals the morphological characteristics of the femurs in PJI mice treated with PBS, vancomycin, alendronate, or both together (scale bar: 200μm). (**B**) Immunohistochemical staining of IL-1β of the femurs of PJI mice bearing implants in the indicated groups (scale bar: 200μm for LPF and 20μm for HPF). (**C**) Immunohistochemical staining of TNF-α of the femurs of PJI mice bearing implants in the indicated groups (scale bar: 200μm for LPF and 20μm for HPF). (**D-E**) Statistics analysis of IHC score of IL-1β and TNF-α (n=5 per groups). (Bar plot displays the means ±SD; ns, not significant, *p < 0.05, ***p < 0.001.)

**Figure S15.** **FC Analysis Results of Immune Cells in the Knee Joint Soft Tissue of PJI Mice, related to Figure 9**

(A) Proportion of PMN-MDSC (Cd45^+^Cd11b^+^Ly6g^+^) in PJI mice in the indicated groups. (B) Proportion of Bregs (Cd45^+^Cd3^-^Cd19^+^Il-10^+^) in PJI mice treated with PBS, vancomycin, alendronate, or both together. (C) Proportion of Gzmb+ CD8+ T cells (Cd45^+^Cd3^+^Cd8b^+^Gzmb^+^) in PJI mice in the indicated groups. (D) Proportion of Infg+ CD8+ T cells (Cd45^+^Cd3^+^Cd8b^+^Infg^+^) in PJI mice in the indicated groups. (E) Proportion of cDCs (Cd45^+^Cd11c^+^MHC-II^+^) in PJI mice in the indicated groups**.**

**Table S1.** **Primers of qRT-PCR**

| Genes | Sequences (5’-3’) |
| --- | --- |
| Mouse ARG1 | F: 5’- ACATTGGCTTGCGAGACGTA -3’  R: 5’- ATCGGCCTTTTCTTCCTTCCC -3’ |
| Mouse PTGS2 | F: 5’- CATCCCCTTCCTGCGAAGTT -3’  R: 5’- CCTCTCCACCAATGACCTGAT -3’ |
| Mouse GAPDH | F: 5’- ACCCTTAAGAGGGATGCTGC -3’  R: 5’- CCCAATACGGCCAAATCCGT -3’ |

**Table S2.** **Information of Reagents and Primary Antibodies**

| Name | Application | Product Information |
| --- | --- | --- |
| Vancomycin | Treatment in vivo | Cat# HY-B0671, MCE |
| Alendronate sodium | Treatment in vivo | Cat# HY-108685, MCE |
| Etidronic acid | Treatment in vivo | Cat# HY-B0302, MCE |
| Zoledronic Acid | Treatment in vivo | Cat# HY-13777, MCE |
| Plerixafor (AMD3100) | Treatment in vivo | Cat# HY-10046, MCE |
| Anti-Ly6g | Treatment in vivo | Clone 1A8; BioXCell |
| Anti-CD22 | Treatment in vitro | Clone Cy34.1; BioXCell |
| Anti-CD72 | Treatment in vitro | AB_394656, Pharmingen |
| Stattic | Treatment in vitro | Cat# HY-13818, Med Chem Express |
| SDF-1 | Treatment in vitro | Cat# HY-P72782, Med Chem Express |
| Colivelin | Treatment in vitro | Cat# HY-P1061, Med Chem Express |
| Collagenase I | Tissue digestion | Cat# 10103578001, Merck |
| Dispase II | Tissue digestion | Cat# D4693, Signa Aldrich |
| Deoxyribonuclease I | Tissue digestion | Cat# abs47047435, Absin |
| PerCP/Cyanine5.5 anti-mouse CD45 | Flow cytometry | Cat# 103132, Biolegend |
| APC/Fire™ 750 anti-mouse/human CD11b | Flow cytometry | Cat# 101262, Biolegend |
| Brilliant Violet 421™ anti-mouse CD11c | Flow cytometry | Cat# 117329, Biolegend |
| Brilliant Violet 605™ anti-mouse Ly-6G | Flow cytometry | Cat# 127639, Biolegend |
| Brilliant Violet 605™ anti-mouse Gr1 | Flow cytometry | Cat# 108439, Biolegend |
| PE/Cyanine7 anti-mouse Ly-6C | Flow cytometry | Cat# 128017, Biolegend |
| Brilliant Violet 711™ anti-mouse CXCR4 | Flow cytometry | Cat# 146517, Biolegend |
| PE anti-mouse CXCR4 Antibody | Cell sorting | Cat# 146505, Biolegend |
| APC anti-mouse I-A/I-E | Flow cytometry | Cat# 107613, Biolegend |
| Brilliant Violet 605™ anti-mouse CD3 | Flow cytometry | Cat# 100237, Biolegend |
| APC/Fire™ 750 anti-mouse CD19 | Flow cytometry | Cat# 115558, Biolegend |
| PE/Cyanine7 anti-mouse CD4 | Flow cytometry | Cat# 116015, Biolegend |
| APC anti-mouse CD8b | Flow cytometry | Cat# 126613, Biolegend |
| PE/Dazzle™ 594 anti-human/mouse Granzyme B Recombinant | Flow cytometry | Cat# 372215, Biolegend |
| Brilliant Violet 785™ anti-mouse IFN-γ | Flow cytometry | Cat# 505837, Biolegend |
| FITC anti-mouse IL-10 | Flow cytometry | Cat# 505006, Biolegend |
| APC anti-mouse CD274 Antibody | Flow cytometry | Cat# 124311, Biolegend |
| PE anti-Ahr | Flow cytometry | Cat# 694503, Biolegend |
| Brilliant Violet 421™ anti-mouse Tim-1 | Flow cytometry | Cat# 119507, Biolegend |
| Zombie Aqua™ Fixable Viability Kit | Flow cytometry | Cat# 423102, Biolegend |
| TruStain FcX™ PLUS (anti-mouse CD16/32) | Flow cytometry | Cat# 156603, Biolegend |
| Cell Activation Cocktail (with Brefeldin A) | Flow cytometry | Cat# 423303, Biolegend |
| Annexin V-FITC Apoptosis Detection Kit | Flow cytometry | Cat# C1062M, Beyotime |
| Cell Proliferation Dye eFluor™ 670 | Flow cytometry | Cat# 65-0840-85, eBioscience |
| Myeloid-Derived Suppressor Cell Isolation Kit | Cell sorting | Cat# 130-094-538, Miltenyi Biotec |
| EasySep Mouse PE Positive Selection Kit II | Cell sorting | Cat# 17656, StemCell |
| EasySep Mouse T-cell Sorting Kit | Cell sorting | Cat# 19851, StemCell |
| EasySep Mouse B-cell Sorting Kit | Cell sorting | Cat# 19854, StemCell |
| Purified anti-mouse Gr-1 Antibody | Immunofluorescence | Cat# 108401, Biolegend |
| Ly-6G (E6Z1T) Rabbit mAb | Immunofluorescence | Cat# 87048, CST |
| CXCR4 Rabbit pAb | Immunofluorescence | Cat# bs-1011R, Bioss |
| CD19 Polyclonal antibody | Immunofluorescence | Cat# 27949-1-AP, Proteintech |
| AHR Polyclonal antibody | Immunofluorescence | Cat# 28727-1-AP, Proteintech |
| TIM1 Monoclonal Antibody | Immunofluorescence | Cat# RMT1-10, Invitrogen |
| Anti-Collagen Type I/COL1A1 Antibody | Immunofluorescence | Cat# PB0980, BOSTER |
| Rabbit polyclonal to Staphylococcus aureus | Immunofluorescence | Cat# ab20920, abcam |
| IL-1 Beta Rabbit pAb | Immunohistochemistry | Cat# bs-0812R, Bioss |
| Anti-TNF Alpha/TNFA Antibody | Immunohistochemistry | Cat# BA0131, BOSTER |
| p-STAT3 (Ser727) Monoclonal antibody | Western blot | Cat# 60479-1-Ig, Proteintech |
| STAT3 Polyclonal antibody | Western blot | Cat# 10253-2-AP, Proteintech |
| p-p65 (Ser536) Monoclonal antibody | Western blot | Cat# 80379-2-RR, Proteintech |
| p65 Monoclonal antibody | Western blot | Cat# 80979-1-RR, Proteintech |
| Arginase-1 Polyclonal antibody | Western blot | Cat# 16001-1-AP, Proteintech |
| PTGS2 Polyclonal antibody | Western blot | Cat# 27308-1-AP, Proteintech |
| Rabbit anti-β-Tubulin Monoclonal Antibody | Western blot | Cat# abs171597, Absin |
| Goat Anti-Rabbit IgG H&L (HRP) | Western blot | Cat# AB205718, Abcam |
